# Majority Voting Versus Learned-Threshold Score Aggregation for Verifying IoT Security Events Under Non-IID Heterogeneity

**DOI:** 10.3390/s26216969

**Published:** 2026-11-04

**Authors:** Ivan Ortiz-Garces, Fernando Molina-Granja, Rommel Gutierrez, Fernando Vela-Espin

**Affiliations:** 1 Escuela de Ingeniería en Ciberseguridad, Universidad de Las Américas, Quito 170124, Ecuador; rommeljair.gutierrez@udla.edu.ec (R.G.); fernando.vela.espin@udla.edu.ec (F.V.-E.); 2 Facultad de Ingeniería, Universidad Nacional de Chimborazo, Riobamba 060108, Ecuador; fmolina@unach.edu.ec

**Keywords:** IoT intrusion detection, majority voting, threshold learning, detector aggregation, calibration, Non-IID heterogeneity, compromised validators

## Abstract

Intrusion detectors for Internet of Things (IoT) systems are usually trained per device or site, so a verification layer must combine alerts from detectors built on different populations and base rates. This study compares two ways of turning such alerts into a decision: majority voting with a fixed threshold, and score aggregation with a threshold learned on labeled calibration data. Seven detectors trained separately on TON_IoT device populations voted on 56,000 shared events, and each rule was compared with the detector local to the event through an amplification factor *A*. Fixed-threshold majority voting amplified error in the point estimate (A=1.12 for 1−MCC), whereas learned-threshold aggregation reduced it (A≈0.62); device-level intervals, however, included neutrality. An equal-weight score with a learned threshold matched reliability-, margin-, and hybrid-weighted rules, so the improvement came from threshold placement rather than from weighting. In this setting, about 500 labeled target events sufficed to place the threshold, whereas source-domain reliability estimates were biased by up to 0.22 although their 95% sampling half-width was at most 0.011. Gains concentrated in devices with weak local detectors and shrank under natural prevalence. Under degraded validators, fixed-threshold voting was the most fragile rule. A synthetic vote layer overstated amplification, and a federated detector showed a shared threshold collapsing under domain shift.

## 1. Introduction

Intrusion detection in Internet of Things (IoT) deployments is rarely a single-model problem. Devices differ in protocol, sensing modality, traffic volume, and attack exposure, and this heterogeneity is a recognized barrier to securing these ecosystems [1]. In practice, each device family, gateway, or site often runs its own detector, and downstream components must decide how to combine alerts from detectors that were trained on different populations and that operate at different base rates. Federated learning is one way to train such detectors without centralizing raw data [2,3], and Byzantine fault-tolerant protocols provide a classical vocabulary for reconciling conflicting reports from unreliable observers [4,5]. Whatever the architecture, a verification layer that receives several alerts about the same event must eventually apply a decision rule, and the simplest and most common rule is a majority vote with a fixed threshold.

A fixed threshold is a strong assumption under non-independent and non-identically distributed (non-IID) heterogeneity. Detectors are commonly evaluated by discrimination, often under the implicit assumption that a model that ranks events accurately will also produce probabilities that match empirical frequencies and a decision threshold that remains valid elsewhere [6]. Verification layers built on such detectors are, in turn, usually evaluated for fault tolerance under the assumption that the aggregated votes represent comparable evidence. When the detectors transfer their operating points to populations with different base rates, some of them vote positive for most events and others almost never do, and a majority threshold of one half has no principled reason to sit at a useful operating point. The alternative is to treat the committee output as a score, the weighted or unweighted fraction of positive votes, and to learn the decision threshold on labeled calibration data.

This study is a comparative evaluation of these two decision rules: fixed-threshold majority voting and learned-threshold score aggregation. Three features make the comparison difficult to settle with synthetic experiments alone. First, the two rules usually differ in two respects at once: published comparisons pit an unweighted majority with a fixed threshold against a weighted score with a learned threshold, so the effect of weighting is confounded with the effect of threshold placement. Second, the votes of separately trained detectors are not independent, and the benefit of aggregation is not uniform across devices, so a pooled metric computed on a convenience sample of events can hide both gains and losses. Third, when votes are generated synthetically from an assumed reliability conditioned on the true label, part of the observed behavior may arise from the generation procedure itself [7].

We therefore compare the two rules on a committee of seven detectors trained separately on seven distinct IoT device populations and evaluated on a shared pool of 56,000 events. The reference for every comparison is the detector local to the device that produced the event, and an amplification factor expresses whether a rule increases or reduces its error. The contribution is empirical and diagnostic rather than architectural; we do not propose a new detection model. The primary contribution is a controlled comparison in which the fixed threshold of majority voting is replaced by a learned threshold while the votes are kept fixed, and in which an equal-weight score with a learned threshold separates threshold placement from vote weighting; reliability-, margin-, and hybrid-weighted scores and the best single validator are evaluated against this baseline. The remaining analyses test how far the comparison generalizes: across devices, under natural prevalence, on held-out devices, as a function of the amount of labeled calibration data available to estimate thresholds and reliabilities, under degraded validators, and with deferral to an imperfect human reviewer. A synthetic verification layer at matched committee size and a separately described federated experiment complete the evidence on fixed-threshold failure.

## 2. Related Work

Federated learning has been repeatedly proposed as a privacy-preserving alternative to centralized intrusion detection in IoT networks [3]. It has recently been extended to the Internet of Medical Things (IoMT), where physiological telemetry is combined with network features under strict privacy constraints [8]. Federated learning performance can deteriorate when client data are non-IID, a condition commonly encountered in IoT deployments, and robust aggregation approaches designed to address this heterogeneity, including adaptive client-clustering schemes [9], continue to be evaluated predominantly through aggregate discrimination metrics. Comparatively little attention has been paid to whether a shared decision threshold remains valid across clients whose base rates may differ by an order of magnitude.

Calibration has received less attention than discrimination in the network-security literature. Calibration-aware federated learning studies have shown that calibration error can increase substantially after federated aggregation even when discrimination metrics remain stable, and have proposed scaling-based corrections [10]. Evidence from other domains indicates that calibration may deteriorate more severely than discrimination under domain shift because a single operating point may not fit the predicted-probability distribution of every domain [11].

Within distributed verification, comprehensive reviews classify Byzantine fault-tolerant protocol families according to their guarantees under an assumed proportion of adversarial nodes [5]. Studies applying these protocols to IoT security generally treat aggregated votes as exogenous inputs rather than as outputs of potentially miscalibrated detectors. In the ensemble-learning literature, weighting classifiers by reliability or probability-based evidence has been reported to outperform unweighted majority voting and the best individual classifier in intrusion-detection tasks [12], and related weighting schemes have been used to integrate evidence from multiple sources in distributed edge systems [13]. Such comparisons typically use an unweighted majority with a fixed threshold as the baseline. They therefore do not separate the contribution of weighting from the contribution of a threshold learned on calibration data, which is the distinction on which the present comparison is built.

Label-flipping poisoning in federated intrusion-detection systems has only recently been examined systematically, and its effectiveness depends strongly on the model family and on the strategy used to select the labels to be flipped [14]. Detector performance also deteriorates as traffic departs from the training distribution [15], whereas threshold manipulation has received less attention as an explicit compromise mechanism. The NIST taxonomy of adversarial machine learning organizes these mechanisms [16]. Finally, reference datasets for intrusion detection may contain labeling artifacts that materially alter reported performance [7], which motivates comparing a synthetic verification layer with a committee of real detectors while recognizing that the two differ in more than the vote-generation procedure.

## 3. Materials and Methods

### 3.1. Study Design and Data Sources

**Figure 1:**
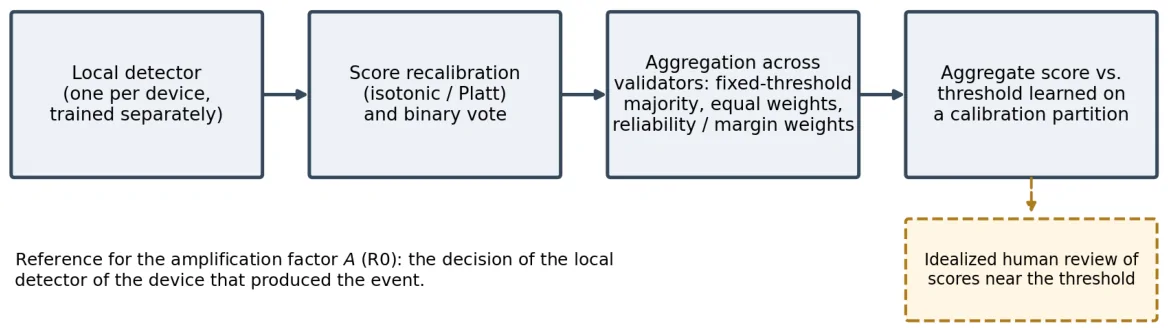
Pipeline of the committee analysis: from local detectors to an aggregate decision under a fixed or learned threshold, with an optional deferral to human review. Solid boxes and arrows show the sequential stages of the analysis; the dashed box and arrow show the optional deferral of scores near the threshold to an idealized human reviewer.

The design compares decision rules applied to the same votes, so that differences between rules cannot be attributed to differences in the detectors. Figure 1 summarizes the pipeline. Each local detector produces a raw score that is converted into a binary vote at its own operating point and recalibrated into a probability; the votes are aggregated by one of the rules; the aggregate score is compared with a fixed or learned threshold; and, optionally, events close to the threshold are deferred to a human reviewer. Every learned threshold was estimated on a dedicated calibration partition and evaluated on a disjoint evaluation partition, and no threshold was adjusted using the data employed to report performance. All uncertainty intervals are two-sided 95% intervals.

The primary committee was built from device-level raw telemetry of the TON_IoT dataset, which provides seven IoT device populations: a smart refrigerator, a GPS tracker, a garage door controller, a Modbus industrial control channel, a motion-activated light, a thermostat, and a weather station. All populations originated from the same collection campaign but were kept separate during feature extraction and training. Table 1 summarizes the population size, natural anomaly rate, and number of attack types after records with unparsable timestamps were removed. Five additional host and network populations (two Linux hosts, two Windows hosts, and one network-flow fragment) were verified as distinct captures and evaluated separately. Because they transferred little information to the IoT pool (pooled AUC between 0.44 and 0.52) and three of them relied on administrative identifiers as their primary signal, they were excluded from the primary analysis and are reported, together with a committee-size grid, only in the Supplementary Material.

The shared evaluation pool contains 56,000 events, 8000 sampled at random from the chronological test segment of each device. Every event of the pool is scored by all seven validators, including those trained on other device types, and the local detector of the originating device serves as the reference. The design is therefore a cross-checking layer that combines non-IID heterogeneity with cross-modality transfer, which limits the conclusions to cross-checking across device types. Because attacks in TON_IoT are concentrated toward the end of each capture, the test segments, and hence the pool, contain a much larger proportion of attacks (0.604) than the natural anomaly rates (0.127–0.224). Results on the pool therefore describe a high-prevalence regime, and they are also reported after reweighting to natural device sizes and natural anomaly rates.

Two further data resources were used only for contrast. The complementary federated experiment uses an integrated hybrid dataset of 113,477 behavioral traces distributed across ten clients and derived from six sources: Edge-IIoTset, a synthetic patient-monitoring flow with injected alerts, and four TON_IoT subsets. It was partitioned into an IoT domain (63,477 traces) and an IoMT domain (50,000 traces) with 1675 aggregated statistical features per trace. A pre-existing synthetic verification dataset containing 100,000 events and votes from 21 validators, generated according to assigned reliability parameters, was retained exclusively as a contrast arm for fixed-threshold majority voting.

### 3.2. Behavioral Feature Space and Validator Construction

A committee member must be able to score an event regardless of the device from which it originated, even though the raw-data schemas differ. Each validator was therefore trained on a device-agnostic behavioral feature space computed causally from current and preceding observations. The feature space comprised the interarrival time and event-burst rate within a 60 s window; the *z*-score, first difference, deviation from a causal moving average, and moving standard deviation of the primary numerical signal; normalized absolute deviation; number of available numerical signals; state-change indicator and state rarity computed from categorical fields; and current-state burst length. The primary signal was the first numerical column of each device’s raw schema or, when none was numerical, the most frequent categorical column, ordinally encoded by its frequency rank in the calibration partition, as Table 2 lists for each population. This eleven-dimensional representation does not depend on a specific protocol or sensor field.

Time-of-day and day-of-week variables were evaluated during feature-space construction through an ablation in which auxiliary detectors were trained using only the calendar variables and, separately, the complete feature space without them. The calendar variables were excluded because they predicted attack occurrence without representing device behavior: attacks were concentrated within a narrow calendar interval shared by all seven devices. Interarrival-time and burst-rate variables were retained because they are operationally related to denial-of-service and scanning activity, although part of their predictive signal may also reflect synchronization within the collection campaign rather than local device behavior alone.

Each validator was trained with a histogram-based gradient-boosting classifier (200 iterations, learning rate 0.1, minimum of 40 samples per leaf, $L_{2}$ coefficient 1.0), using the same configuration for every validator so that performance differences primarily reflect population heterogeneity. Each population was partitioned chronologically into training, calibration, and test segments. Because attacks were concentrated toward the end of each capture, the training segment ended at the temporal midpoint between the first recorded attack and the end of the capture, the calibration segment covered the following quarter of that interval, and the remaining observations formed the test segment. The calibration segments contained between 15,773 and 74,061 events, of which between 2466 (thermostat) and 20,233 (weather station) were attacks. For every source population, none of its events in the shared pool originated from its training or calibration segments, and data from the other populations were structurally disjoint from that validator’s training and calibration data.

“Separately trained” means that each validator was trained on its own data, without access to the training data or scores of the other validators; it does not imply statistical independence among their errors, and the components are therefore described as trained separately on distinct populations rather than as independent observers.

### 3.3. Decision Rules

Let $r_{j}\left(e\right)$ denote the raw score of validator j∈{1,…,7} for event *e*. On its own calibration segment, each validator selected the raw-score threshold that maximized the Matthews correlation coefficient (MCC). Because natural anomaly rates differ across populations, this operating point was transferred to the pool as an alert budget: the proportion $q_{j}$ of calibration events classified as positive was retained, and the pool threshold $t_{j}$ was set to the $\left(1-q_{j}\right)$ quantile of the validator’s raw scores on the pool. The binary vote is

(1) $$\begin{array}{cc} {\mathrm{vote}}_{j}\left(e\right) & =1\;\left[r_{j}\left(e\right)>t_{j}\right]. \end{array}$$

Raw scores were also recalibrated on the validator’s own calibration segment by isotonic regression and, as a variant, by Platt scaling. Writing $g_{j}$ for the recalibration map, the recalibrated score and the vote boundary on the probability scale are

(2) $$\begin{array}{c} s_{j}\left(e\right)=g_{j}\;\left(r_{j}\left(e\right)\right),\;\tau_{j}=g_{j}\;\left(t_{j}\right). \end{array}$$

The map $g_{j}$ is fitted on the validator’s own calibration segment, whereas $t_{j}$ is the quantile that transfers its alert budget to the pool, so $\tau_{j}$ is the representation of the transferred threshold on the calibrated scale, obtained by evaluating the source-domain map at $t_{j}$. Because the isotonic map is non-decreasing, every positive vote satisfies $s_{j}\left(e\right)\geq \tau_{j}$ and every negative vote satisfies $s_{j}\left(e\right)\leq \tau_{j}$. The map is not strictly increasing, however: it is constant on plateaus, so raw scores on both sides of $t_{j}$ can share the calibrated value $\tau_{j}$, and the vote cannot in general be recovered from $s_{j}\left(e\right)$ by a strict inequality. On the pool, the plateau that contains $\tau_{j}$ held between 1% and 13% of the scores for four validators, and 54%, 84%, and 99.9% for the garage-door, thermostat, and GPS-tracker validators. The vote is therefore always defined on the raw score by Equation (1), and $\tau_{j}$ is used only to measure margins. Platt scaling is strictly monotone, but its slope can be negative, in which case the side of $\tau_{j}$ on which a positive vote lies is reversed. The boundary $\tau_{j}$ is generally not 0.5: on the pool it ranged from 0.10 (thermostat) to 0.89 (refrigerator).

All rules combine the seven votes into an aggregate score and compare it with a threshold θ,

(3) $$\begin{array}{cc} \mathrm{agg}(e) & =\frac{\sum_{j}w_{j}\left(e\right)\;{\mathrm{vote}}_{j}\left(e\right)}{\sum_{j}w_{j}\left(e\right)},\;\hat{y}\left(e\right)=1\;\left[\mathrm{agg}(e)>\theta \right], \end{array}$$

and differ only in the weights $w_{j}\left(e\right)$ and in how θ is chosen. Rule R1 is unweighted majority voting, with $w_{j}\left(e\right)=1$ and the fixed threshold θ=0.5, so that four of seven positive votes are required. The equal-weight baseline (EW) uses the same unweighted score, the fraction of positive votes, but learns θ on calibration data; R1 and EW therefore differ only in threshold placement. Rule R2 weights each vote by a static reliability $w_{j}\left(e\right)=\rho_{j}$, following reliability-weighted evidence aggregation in distributed systems [13]. Rule R3 weights each vote by an event-specific decision margin $w_{j}\left(e\right)=m_{j}\left(e\right)$, and the hybrid Rule R4 uses $w_{j}\left(e\right)=\rho_{j}\;m_{j}\left(e\right)$, motivated by evidence that classifier-level reliability and instance-specific evidence can jointly outperform either component alone [12]. The decision margin measures how far the recalibrated score lies from the validator’s own vote boundary, normalized separately on each side so that it spans [0,1]:

(4) $$\begin{array}{cc} m_{j}\left(e\right) & =\left\{\begin{array}{cc} \frac{s_{j}\left(e\right)-\tau_{j}}{1-\tau_{j}}, & s_{j}\left(e\right)\geq \tau_{j},\\ \frac{\tau_{j}-s_{j}\left(e\right)}{\tau_{j}}, & s_{j}\left(e\right)<\tau_{j}. \end{array}\right. \end{array}$$

The margin is 0 for a score on the boundary and 1 for a score at the extreme of the side on which the vote was cast; it reduces to $2|s_{j}\left(e\right)-0.5|$ when $\tau_{j}=0.5$. Measuring it from $\tau_{j}$ rather than from 0.5 ensures that a positive vote is never weighted by its distance below 0.5, which would reward a score that the validator itself places on the negative side of a probability of one half. Recalibration does not guarantee that this distance predicts whether the vote is correct, and we test that relationship empirically; R3 is therefore described as margin-based weighting and the margin is not interpreted as validated confidence.

The reliability of validator *j* is its balanced accuracy on its own calibration segment,

(5) $$\begin{array}{cc} \rho_{j} & =\frac{1}{2}\left({\mathrm{TPR}}_{j}+{\mathrm{TNR}}_{j}\right), \end{array}$$

which is bounded in [0,1] with 0.5 representing chance-level performance. A variant in which $\rho_{j}$ was measured against the evaluation labels was used only as an upper-bound benchmark and not as a deployable rule. MCC was not used as a weight because it can be negative or undefined; a sensitivity analysis with the chance-centered weight $\rho_{j}^{+}=\max\left(0,2\rho_{j}-1\right)$ changed the MCC of R2 and R4 by less than 0.005.

For EW, R2, R3, and R4, the aggregation threshold was learned on one half of the shared pool, using a split stratified by device, and evaluated on the disjoint remaining half (n=28,000 each):

(6) $$\begin{array}{cc} \hat{\theta } & =\underset{\theta \in \Theta }{\arg\;\max}\;\mathrm{MCC}\;\left({\left\{\left(y\left(e\right),\;1\;\left[\mathrm{agg}(e)>\theta \right]\right)\right\}}_{e\in C}\right), \end{array}$$

where C is the calibration half and Θ contains the 1st to 99th percentiles of the aggregate score on C. Two further references complete the comparison. Rule R0 is the reference decision: for each event, the out-of-sample vote of the validator trained on the device that produced it. The best-single-validator baseline (BV) applies to every event the committee member with the highest MCC on the calibration half. BV uses one detector, whereas EW and R2–R4 combine all seven, so BV isolates the benefit of aggregating at all, and EW isolates the benefit of weighting relative to equal weights.

### 3.4. Evaluation Protocol and Uncertainty Quantification

Following the principle that an aggregation mechanism should be evaluated relative to an explicit baseline rather than solely through absolute performance [5], we define the amplification factor of rule R for the error measure *m* as

(7) $$\begin{array}{cc} A_{m}\left(R\right) & =\frac{{\mathrm{Err}}_{m}\left(R\right)}{{\mathrm{Err}}_{m}\left(R0\right)}. \end{array}$$

Values above 1 indicate that the rule is worse than the local detector. We calculated *A* using 1−MCC, the false-negative rate, and the Brier score. The primary uncertainty quantification is a device-level block bootstrap in which the seven device populations are resampled with replacement (10,000 replicates); because the generalization target is the population of IoT devices rather than the individual event, this is the conservative unit. For comparison, event-level intervals were obtained with a nonparametric multinomial bootstrap of the eight joint categories defined by the observed label, the R0 decision, and the decision of the rule under comparison, stratified by device (10,000 replicates); these intervals may be too narrow when temporal or session dependence is not captured by the device stratum. Brier-score intervals used a normal approximation that ignores the positive dependence between predictions on the same events and is therefore conservative for between-rule comparisons. Differences in *A* between rules were evaluated with paired device-level bootstrap draws; McNemar’s test was not used because it assesses classification accuracy rather than MCC. Calibration was quantified with the Brier score and the expected and maximum calibration errors computed with 15 equal-mass bins. All stochastic analyses used a fixed random seed.

Four analyses address the limits of a pooled, convenience-sampled evaluation. First, results were reweighted to natural device sizes and natural anomaly rates. Second, a leave-one-device-out analysis learned each rule’s threshold on the calibration halves of six devices and applied it to the evaluation half of the seventh, to test whether learned thresholds transfer to unseen devices. Third, a leave-one-validator-out analysis re-estimated each rule after removing one committee member at a time. Fourth, the dependence of the reliability estimates and thresholds on the amount of labeled calibration data was examined in two settings. In the source domain, where each $\rho_{j}$ is estimated from $n_{j}^{+}$ attacks and $n_{j}^{-}$ benign events of the validator’s own calibration segment, the estimated standard error of the balanced accuracy, under independent binomial sampling within each class, and its upper bound are

(8) $$\begin{array}{cc} \mathrm{SE}\left({\hat{\rho }}_{j}\right) & =\frac{1}{2}\sqrt{\frac{{\hat{\mathrm{TPR}}}_{j}\left(1-{\hat{\mathrm{TPR}}}_{j}\right)}{n_{j}^{+}}+\frac{{\hat{\mathrm{TNR}}}_{j}\left(1-{\hat{\mathrm{TNR}}}_{j}\right)}{n_{j}^{-}}}\\ & \leq \frac{1}{4}\sqrt{\frac{1}{n_{j}^{+}}+\frac{1}{n_{j}^{-}}}, \end{array}$$

where the bound follows from p(1−p)≤1/4. We report 1.96 times the bound as the largest possible 95% sampling half-width and compare it with the transfer gap between the source-domain estimate and the reliability measured on the pool. In the target domain, *n* labeled events, with *n* equal to 25, 50, 100, 200, 500, 1000, 2000, 5000, or 14,000, were drawn without replacement from the calibration half of the pool (1000 draws per *n*). In each draw, the reliabilities were re-estimated on the *n* events, the thresholds of EW and of R2 with these target reliabilities (R2-target) were re-learned on the same events, and both rules were evaluated on the evaluation half. We report the dispersion of ${\hat{\rho }}_{j}$, the stability of the reliability ranking (Kendall’s τ against the ranking obtained on the full calibration half), the largest deviation of any R2 weight share from its full-half value, and the distribution of *A*.

### 3.5. Robustness Analyses: Degraded Validators and Imperfect Human Review

We assessed the sensitivity of the rules to compromised validators by degrading real detectors through three mechanisms grounded in the NIST taxonomy of adversarial attacks against machine learning systems [16], rather than by generating synthetic adversarial votes. A degraded twin was built for each of the seven IoT validators and for each mechanism. The poisoning mechanism, consistent with label-flipping attacks studied in federated intrusion detection [14], retrains the detector after flipping 80% of the positive training labels to negative. Directed flipping targets the minority class and corresponds to an adversary that hides attacks as benign traffic. The obsolescence mechanism, motivated by evidence that detector performance degrades as traffic departs from the training distribution [15], retrains the detector using only the training data available up to the 50th recorded attack, because restricting training to the period preceding the first attack leaves no positive examples. The threshold-bias mechanism keeps the honest model but replaces its decision threshold with the 98th percentile of its calibration scores. Each twin carries its own vote boundary $\tau_{j}$, so the margins of Equation (4) are always measured from the boundary of the vote that was actually cast.

The committee always comprises seven seats, and a compromised seat is the degraded twin of the same source that it replaces, so each seat holds a distinct identity. For each mechanism and each number of compromised seats c∈{0,…,7}, all 7c subsets were enumerated, and we report the mean, minimum, and maximum amplification factor over subsets. This deterministic enumeration is a stress test of the decision rules against specific degradation profiles; it is not a model of an adaptive adversary. In a mixed-mechanism scenario, each compromised seat independently received one of the three mechanisms (300 random assignments per *c*). Aggregation thresholds were re-learned on the calibration half for every committee, and R0 remained the honest local detector. Severity was varied by repeating the enumeration with 50% and 95% directed label flipping, a 30% symmetric random flip, and complete label inversion; with obsolescence cut-offs of 25 and 100 attack samples; and with threshold percentiles of 0.90, 0.95, and 0.99.

Finally, to examine how an operating policy could use a continuous aggregate score, we deferred an event to human review whenever its R3 score lay within a symmetric margin δ of the learned threshold, $|{\mathrm{agg}}_{R3}\left(e\right)-\hat{\theta }|\leq \delta$ [17]. R3 was used because its score is continuous, whereas the EW score takes only eight values. A common simplification assumes that every deferred event is resolved correctly, which yields an upper bound. Here the reviewer’s accuracy a∈{1,0.95,0.90,0.80,0.70} is an explicit parameter: a deferred event receives its true label with probability *a* and the opposite label with probability 1−a, and metrics are computed from expected confusion counts. Margins δ∈{0,0.03,0.06,0.10,0.15,0.20} were evaluated.

### 3.6. Complementary Experiment: Federated Detector Under Non-IID Domains

The federated experiment is separate from the committee analysis. It uses different data and a different model, and it is included for a single purpose: to show, independently of any verification layer, that a decision threshold shared across heterogeneous domains can fail even when discrimination and aggregate calibration remain good. This is the mechanism that makes a fixed 0.5 majority threshold inadequate for a committee whose members have different transferred alert rates, and its results are not combined with those of the committee.

We trained a logistic regression classifier on the ten clients using a manual implementation of Federated Averaging (FedAvg) [18]. Logistic regression was selected because its parameter vectors can be averaged directly in a common weight space, unlike independently trained tree ensembles. Training comprised 25 communication rounds and two local epochs per round, using logistic loss with $L_{2}$ regularization (α=0.001). An aggregate feature representing the anomalous-event ratio was excluded because it formed part of the operational definition of the target label and would constitute target leakage. Missing feature values, representing protocols or modalities absent from a trace’s domain, were encoded as zero. A single global threshold was learned by maximizing MCC on the pooled calibration partitions of the ten clients and was intentionally not customized by client, so as to expose the effect of a genuinely shared operating point.

## 4. Results

### 4.1. Validator Heterogeneity, Calibration, and Error Dependence

Table 3 shows that the seven validators differed markedly in transferred performance on the shared pool. Pooled AUC ranged from 0.51 for the GPS-tracker validator to 0.82 for the motion-light validator, and individual MCC ranged from −0.04 (garage door) to 0.52 (motion light). Transferred alert rates ranged from 2% to 68% of events, a consequence of transferring each validator’s calibration-based alert budget to a pool whose prevalence (0.604) differs from that of the validator’s own population. This dispersion of alert rates is the property that a fixed majority threshold cannot accommodate.

**Figure 2:**
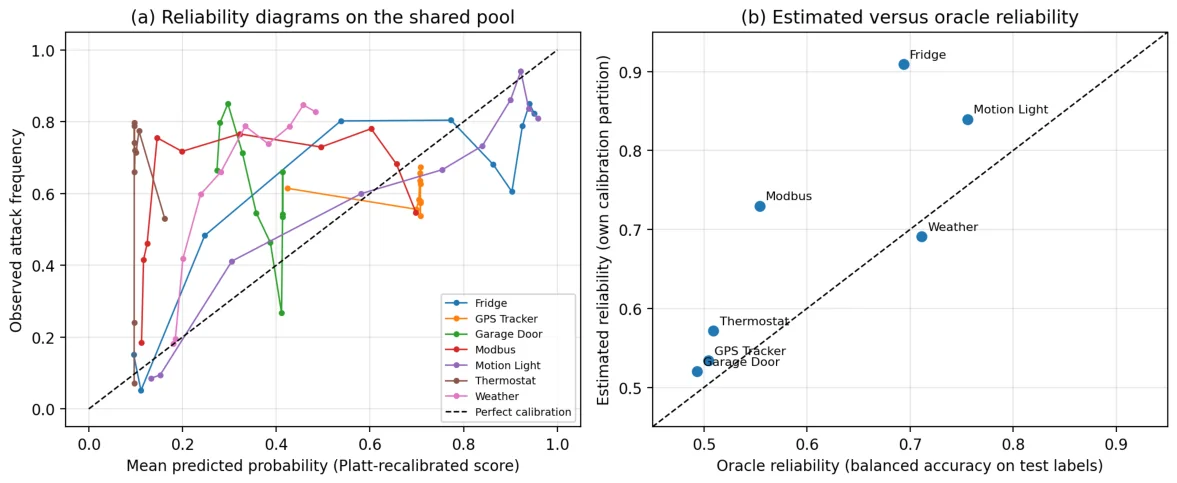
Calibration and transferred reliability of the seven IoT validators on the shared pool: (**a**) reliability diagrams; (**b**) reliability estimated on each validator’s calibration segment versus oracle reliability measured on the pool labels.

In Figure 2a, the reliability diagrams show how calibration transfers to the shared pool. The refrigerator and motion-light validators remain comparatively close to the diagonal at predicted probabilities above 0.8. The thermostat, weather-station, and Modbus validators systematically underestimate risk: the thermostat’s mean predicted probability never exceeds about 0.16, although observed attack frequencies reach 0.8, a pattern expected when validators calibrated on populations with anomaly rates of 0.13–0.22 score a pool whose prevalence is 0.604. The GPS-tracker and garage-door validators exhibit irregular, compressed calibration patterns, indicating unstable calibration transfer beyond their training populations. Figure 2b compares each validator’s reliability estimated on its calibration segment with its oracle reliability measured on the pool labels. Except for the weather-station validator, the estimate exceeds the oracle value, indicating systematic optimism in source-domain estimates; the largest discrepancy occurs for the refrigerator validator (0.91 estimated versus 0.69 oracle).

**Figure 3:**
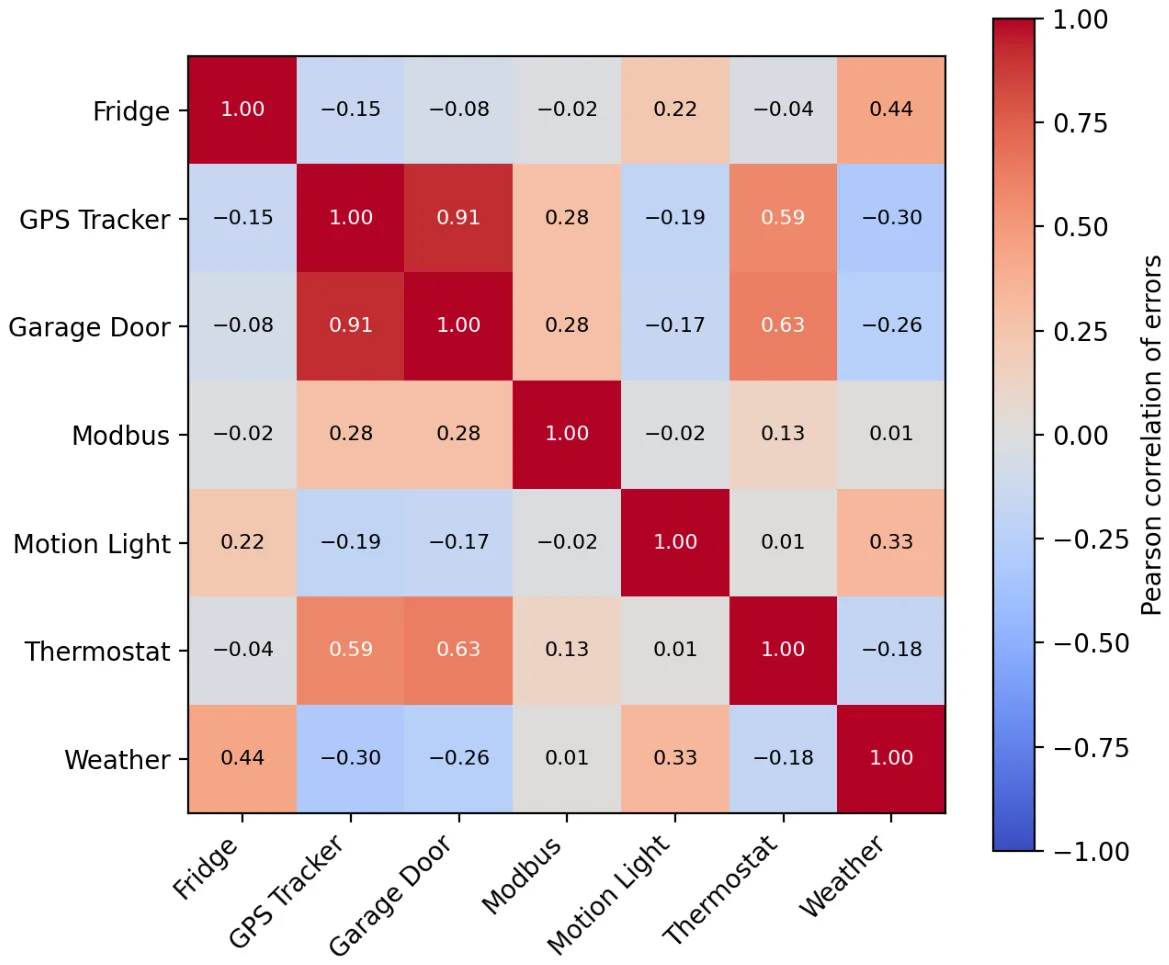
Pairwise correlation of validator errors among the seven IoT validators on the evaluation half of the shared pool.

Training validators separately does not make their errors statistically independent. Figure 3 presents the pairwise error correlations. Across the 21 validator pairs, the median absolute correlation was 0.19, the mean signed correlation was 0.11, and 3 pairs had |r|>0.5, including the GPS tracker–garage door pair (r=0.91). Using the variance-inflation approximation $N_{\mathrm{eff}}=7/\left(1+6\bar{r}\right)$, the seven validators carry the information of about 4.1 independent validators, and an eigenvalue-based estimate gives 4.2. None of the rules adjusts its weights for error dependence, so the nominal committee size overstates the independent evidence.

### 4.2. Fixed-Threshold Majority Voting Versus the Local Detector

The alert-rate dispersion creates a structural asymmetry. A majority in the seven-member committee requires at least four positive votes, whereas only the refrigerator, motion-light, and weather-station validators vote positive for more than 60% of events. Agreement from at least one low-alert-rate validator is therefore needed, and such agreement is infrequent on events that originate outside those validators’ training populations. Table 4 compares R1 with R0 on all 56,000 events.

Recall decreased from 0.483 to 0.309, whereas precision changed only from 0.775 to 0.761. The principal failure of majority voting was therefore not an increase in false positives but a reduced ability to identify attacks. The improvement in Brier score alongside the deterioration in MCC and false-negative rate is not contradictory: the fraction of positive votes preserves information about the degree of committee agreement, whereas its conversion into a binary decision at a fixed threshold discards it. This observation motivates replacing the fixed threshold by a learned one.

The event-level intervals may be too narrow if observations within a device are not exchangeable. Resampling the seven devices as complete blocks left the point estimate essentially unchanged (A=1.12) but widened the 95% interval for 1−MCC to [0.84,1.69], and for the false-negative rate to [0.83,3.63]. Both intervals include the neutral value: the point estimates indicate amplification, but with seven devices it cannot be established at the 95% level, and R1 was below A=1 in 24% of the device-level bootstrap replicates.

**Figure 4:**
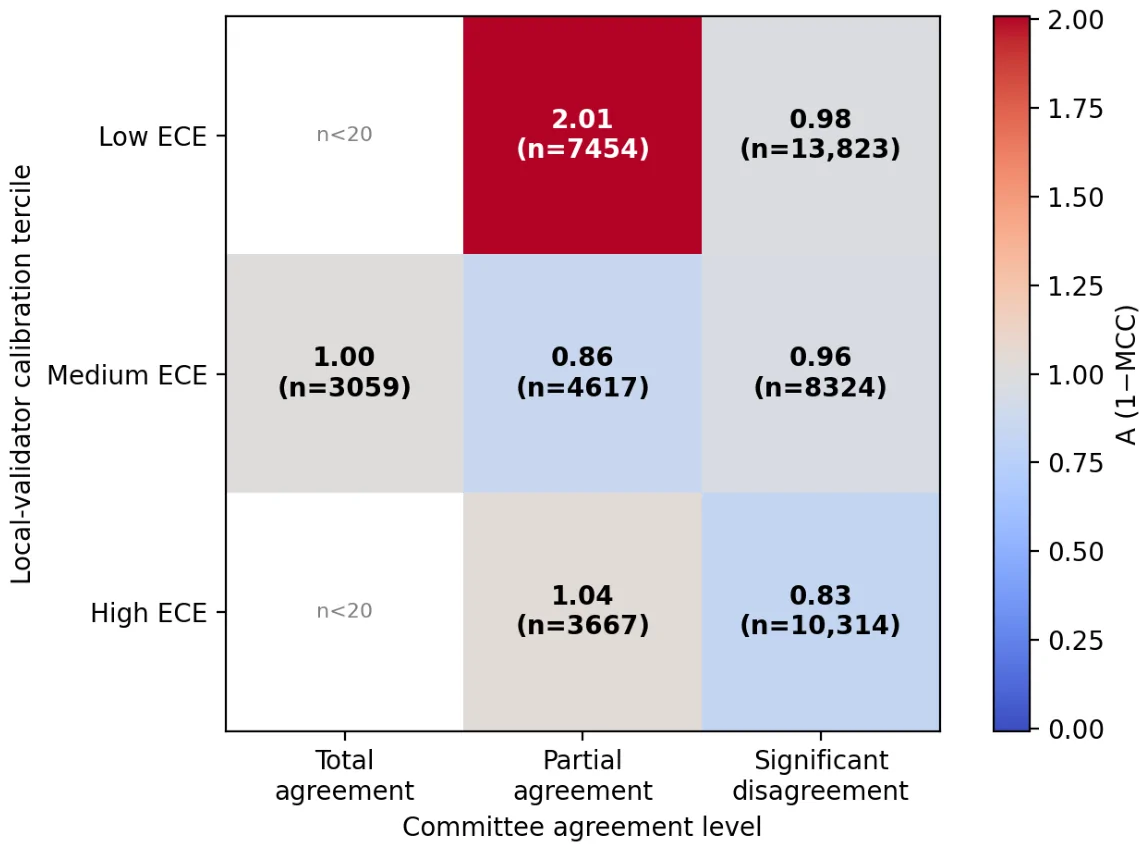
Amplification factor (1−MCC) of fixed-threshold majority voting when crossing the local validator’s calibration tertiles with the committee’s level of agreement.

Figure 4 shows that the pooled point estimate conceals substantial variation. After cross-classifying the local validator’s calibration level (tertiles of expected calibration error) with the committee’s agreement level, the largest amplification occurred under low ECE combined with partial committee agreement (A=2.01, n=7454), and the largest error reduction under high ECE combined with substantial disagreement (A=0.83, n=10,314); the remaining cells ranged from 0.86 to 1.04. One interpretation is that partial committee support can override a comparatively well-calibrated local validator, whereas substantial disagreement can correct a poorly calibrated one. Because the cross-tabulation does not establish which members were correct within individual events, this remains a hypothesis.

**Figure 5:**
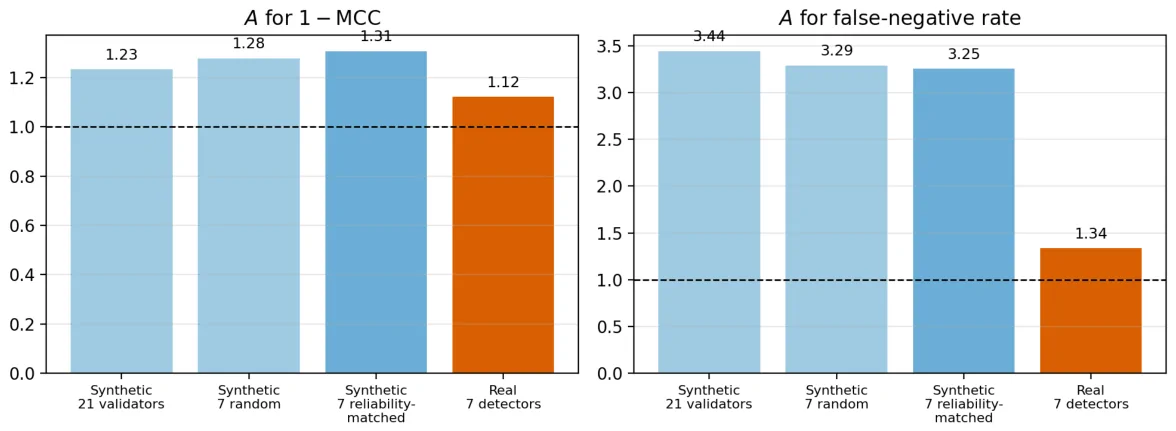
Amplification factor of majority voting for the synthetic layer at full size (21 validators), at matched size (seven validators, random and reliability-matched), and for the real-detector committee.

A synthetic verification layer (21 validators, votes generated from assigned reliabilities conditioned on the true label) reproduced the direction of this effect but not its magnitude: A=1.23 versus 1.12 for 1−MCC, and 3.44 versus 1.34 for the false-negative rate. Because the difference between 21 and 7 validators could confound the comparison, the synthetic analysis was repeated with seven validators, as Table 5 and Figure 5 report. Seven randomly drawn synthetic validators gave a median A=1.28 (95% range of draws [1.24,1.35]) for 1−MCC and 3.29 ([3.16,3.40]) for the false-negative rate; seven synthetic validators whose generating reliabilities were matched one-to-one to the estimated reliabilities of the real validators gave 1.31 and 3.25. Committee size therefore does not explain the gap: even at matched size, the synthetic layer overstated the amplification of fixed-threshold majority voting by a factor of about 2.4 for the false-negative rate. The comparison cannot isolate whether label-conditioned vote generation, the dependence among validators, or the heterogeneity of their alert rates produces the gap, but it shows that conclusions about fixed-threshold voting drawn from the synthetic layer alone would not transfer to the real-detector committee.

### 4.3. Learned-Threshold Score Aggregation and the Contribution of Weighting

Table 6 reports all rules on the held-out evaluation half (n=28,000; anomaly rate 0.603). Replacing the fixed threshold of R1 by a threshold learned on the calibration half, while keeping the same unweighted votes, reversed the outcome. The equal-weight baseline raised recall from 0.309 to 0.942 and gave A=0.62 for 1−MCC. Its learned threshold was 1/7, so EW alerts when at least two of the seven validators vote positive, in contrast with the quorum of four required by majority voting. The weighted rules reached the same level: A=0.62 for reliability weighting (R2), 0.63 for margin weighting (R3), and 0.62 for the hybrid (R4). Paired device-level bootstrap differences between each weighted rule and EW were small and their intervals included zero (R2: −0.003, [−0.010,0.004]; R3: +0.011, [−0.043,0.083]; R4: −0.002, [−0.060,0.052]). The best single validator (motion light, selected on the calibration half) reached A=0.66, a value not distinguishable from those of the committee rules (paired difference to EW +0.036, [−0.184,0.270]). The change that separates the two families of rules is therefore the learned threshold applied to an aggregate score, not the weighting.

The rules with equal MCC did not operate at the same point. EW and R2 favored recall (0.94–0.95, precision 0.76), whereas the margin-weighted rules R3 and R4 traded recall for precision (recall 0.83, precision 0.81–0.82). All learned-threshold rules improved on R0 in the point estimate, but the device-level intervals for 1−MCC reached or exceeded A=1 (upper limits 1.06–1.10), so error reduction relative to the local detector is not established across devices at the 95% level. For the false-negative rate, the intervals of EW and R2 lie well below 1 (upper limits 0.27 and 0.23), whereas those of R3 and R4 reach it (1.07 and 1.06) because of their lower recall. In the device-level bootstrap, every learned-threshold rule was below A=1 in 95% of replicates, compared with 24% for R1. With Platt instead of isotonic recalibration, R3 reached an MCC of 0.556 (A=0.61) at a recall of 0.93; the Platt map had a negative slope for the GPS-tracker and garage-door validators, whose calibration AUC was below 0.5, so their margins measure distance from the boundary in the opposite direction of the raw score. Replacing estimated by oracle reliabilities changed the MCC of R2 by 0.0005 and that of R4 by 0.003.

Whether the decision margin carries information about vote correctness was tested on all 196,000 validator–event pairs of the evaluation half. A total of 37% of votes had a margin of exactly zero because their recalibrated score lay on the isotonic plateau that contains the vote boundary, and these votes were wrong in 60% of cases. Ordering all votes by margin, the error rate in the five quintiles was 0.59, 0.54, 0.38, 0.38, and 0.25, and the Spearman correlation between margin and vote error was −0.24; it was negative for six of the seven validators and positive only for Modbus (0.15). The corrected margin is therefore a weak but correctly oriented indicator of vote correctness. An ablation clarifies how it acts within R3. Permuting the seven margins among validators within each event, which preserves their distribution but breaks the link between a vote and its own margin, reduced MCC from 0.542 to 0.430; replacing each event-specific margin by the validator’s mean margin gave 0.546, and replacing margins by within-event ranks gave 0.542. Most of the effect of R3 is therefore a structural reweighting across validators: the motion-light, Modbus, and refrigerator validators jointly received 78% of the weight, whereas the GPS-tracker and thermostat validators, whose votes lie mostly on or near their boundary plateau, received almost none.

**Figure 6:**
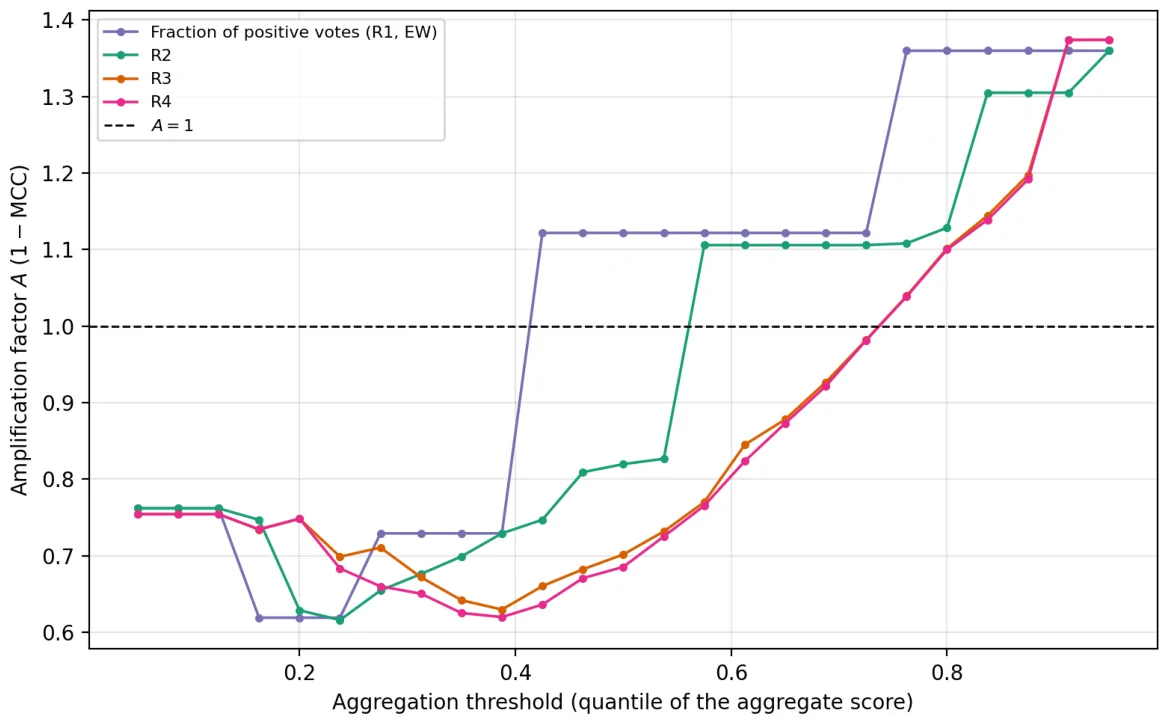
Amplification factor (1−MCC) as a function of the aggregation threshold, expressed as a quantile of each aggregate score, on the evaluation half. The unweighted score is the fraction of positive votes used by R1 (fixed threshold 0.5) and EW (learned threshold). The horizontal reference at A=1 denotes the boundary between error reduction and amplification.

Figure 6 shows why threshold placement dominates. Sweeping the threshold over the range of each aggregate score moved every rule between A≈0.62 and values above 1.3. For the unweighted score, the fixed threshold of 0.5 falls on the plateau at A=1.12, and the learned threshold of 1/7 falls at the minimum. The margin-weighted scores of R3 and R4 changed smoothly with the threshold, whereas the unweighted and reliability-weighted scores changed in steps, so a misplaced threshold costs less with a continuous score; at their respective optima, however, the rules did not differ.

The leave-one-validator-out results in Table 7 show that the committee is not uniformly sensitive to its members. Removing the motion-light validator lowered the MCC of the learned-threshold rules from about 0.55 to 0.46–0.50, and removing the refrigerator validator lowered it to 0.48–0.52, whereas removing the GPS-tracker or garage-door validator changed it by less than 0.01. R1 was far more erratic, ranging from −0.03 to 0.20 depending on which validator was removed. The learned-threshold rules are thus driven by the two or three validators with transferable signal, consistent with their weight shares.

### 4.4. Heterogeneity Across Devices, Natural Prevalence, and Unseen Devices

**Figure 7:**
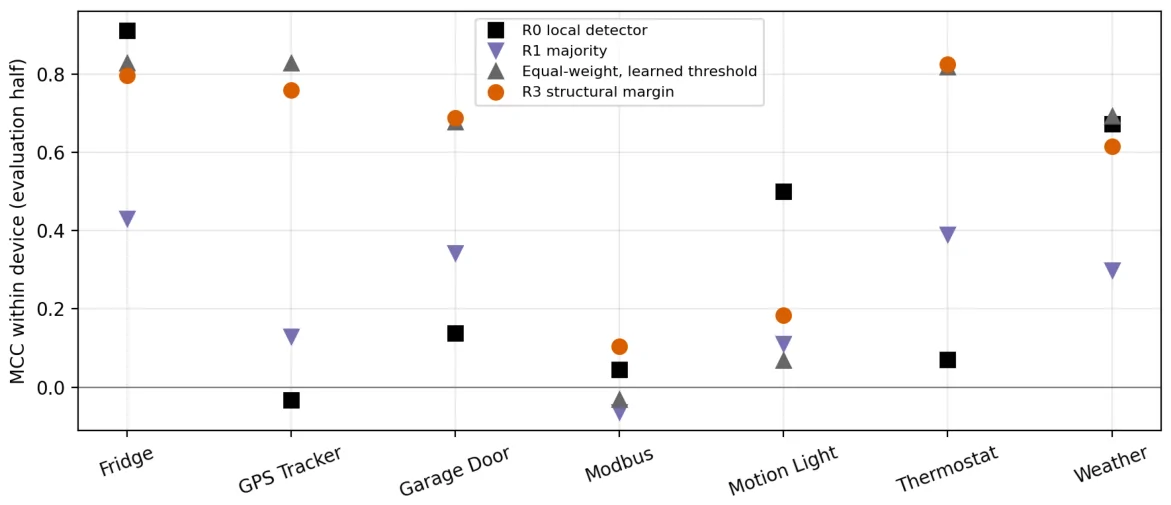
Within-device MCC of the local detector (R0), fixed-threshold majority voting (R1), the equal-weight baseline with a learned threshold, and R3 on the evaluation half of the shared pool.

Table 8 and Figure 7 show that the pooled gains hide strong differences between devices. EW improved on R0 for four of the seven devices: GPS tracker (MCC from −0.03 to 0.83), thermostat (0.07 to 0.82), garage door (0.14 to 0.68), and weather station (0.67 to 0.69). It worsened the other three. The refrigerator, whose local detector was already strong (MCC 0.91), fell to 0.83; the motion-light device fell from 0.50 to 0.07, even though its own validator is the best single committee member on the pool; and the Modbus channel remained near zero. The weighted rules showed the same pattern with small shifts: R3 and R4 lifted the motion-light and Modbus devices slightly (to 0.15–0.18 and 0.10–0.11) at some cost for the GPS tracker (0.75–0.76), and R4 improved five devices. R1 improved three. Aggregation therefore transfers the strength of the best validators to devices whose local detectors are weak, at the cost of degrading devices whose local detectors are good, and a policy that applied the committee decision to every device would need to account for this trade-off, for example by retaining the local decision where the local detector has demonstrated high reliability.

The shared pool has a prevalence of 0.604, far above the natural rates of 0.127–0.224. Table 9 therefore reweights evaluation events either to the natural device sizes or to both natural sizes and natural anomaly rates. Under natural size and prevalence, MCC fell for all rules, and the amplification factors moved toward neutrality with device-level intervals that reach 1. The ordering between the two families was preserved: R1 stayed near neutrality (A=1.07), whereas every learned-threshold rule stayed below it. The size of the benefit, however, shrank from about 38% on the pool to 17–18% for EW, R2, and BV (A=0.82–0.83) and 25–28% for R3 and R4 (A=0.75 and 0.72). The advantage of the margin-weighted rules in this setting follows from their operating point rather than from a better ranking: when attacks are rare, each false positive costs more MCC, and R3 and R4 were already operating at higher precision. The thresholds were learned at a prevalence of 0.60, so the result also indicates that a threshold learned at one prevalence is too permissive at a lower one. We therefore regard the pooled results as describing a high-prevalence regime and not as expected field performance.

The learned thresholds were estimated on calibration events from the same seven devices that are later evaluated. To test transfer, Table 10 learns each threshold on the calibration halves of six devices and applies it to the evaluation half of the held-out device. The device-balanced pooled MCC was 0.549 for EW, 0.509 for R2, 0.471 for R3, and 0.541 for R4, compared with 0.272 for R0, giving amplification factors of 0.62, 0.67, 0.73, and 0.63. EW was unaffected because its threshold remained at 1/7 whichever device was held out, so the low-quorum rule does not depend on the held-out device’s own events. The weighted rules were less stable. R2 fell to −0.16 on the motion-light device, consistent with reliability weights that are optimistic and device-specific, and R3 fell to 0.24 on the GPS tracker (0.76 with in-sample thresholds), because its continuous score places the learned threshold more finely and therefore depends more on which devices supply the calibration events. The per-device pattern of gains for weak local detectors and losses for strong ones persisted under leave-one-device-out thresholds.

### 4.5. Calibration-Set Size and the Stability of Reliability and Threshold Estimates

Reliability weights and learned thresholds are both estimated from labeled calibration data, so their stability depends on how much of it is available. Two sources of error must be distinguished: sampling error, which decreases with the size of the calibration set, and transfer bias, which arises because reliability is estimated on a validator’s own population and applied to another one. Table 11 compares them for the source-domain estimates used by R2 and R4. The calibration segments contained between 2466 and 20,233 attacks, so the 95% sampling half-width of every ${\hat{\rho }}_{j}$ was at most 0.004–0.011. The difference between the source estimate and the reliability measured on the pool was larger than this half-width for all seven validators, and reached 0.22 for the refrigerator and 0.18 for the Modbus validator. The uncertainty that matters for the source-domain weights is therefore transfer bias, which more calibration data from the same population cannot reduce.

**Figure 8:**
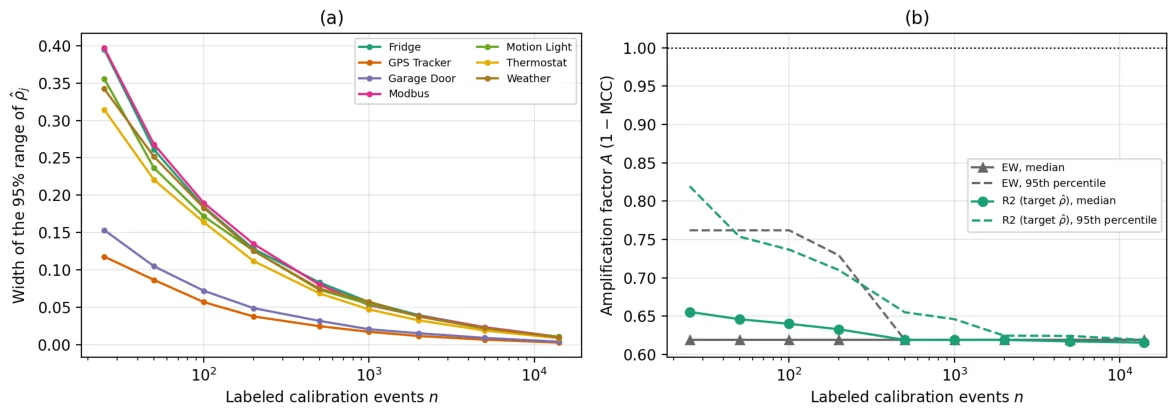
Effect of the number *n* of labeled target events on (**a**) the width of the 95% range of each validator’s estimated reliability and (**b**) the median and 95th percentile of the amplification factor of EW and of R2 with target reliabilities (1000 draws per *n*).

Estimating reliabilities on labeled events from the target population removes the transfer bias but makes sampling error the limiting factor. Table 12 reports the subsampling analysis. The standard deviation of ${\hat{\rho }}_{j}$, averaged over validators, was 0.076 with n=25 labeled events, 0.037 with n=100, 0.016 with n=500, and 0.005 with n=5000, and Figure 8a shows that the width of the 95% range for the most variable validators fell from about 0.40 to below 0.06 at n=1000. Validators whose votes are rare, the GPS tracker and the garage door, had the narrowest ranges because their true-positive and true-negative rates lie close to 0 and 1. The ranking of the validators by reliability was unstable for small *n*: the median Kendall τ with the full-half ranking was 0.62 at n=25, 0.81 at n=100–200, and 0.90 at n=500–1000, and it reached 1 only from n=2000. Despite this, the R2 weights were stable, because normalizing reliabilities that lie between 0.49 and 0.75 compresses their differences: the largest deviation of any weight share from its full-half value had a median of 2.7 percentage points at n=25 and 0.9 points at n=200.

Figure 8b shows that the consequences for the decision were limited by the threshold rather than by the weights. With n=50, EW recovered the full-half threshold of 1/7 in 67% of draws and its median amplification factor was already 0.62; with n=200 the proportion was 92%, and from n=500 the threshold was recovered in every draw or all but a few, so the 95th percentile of *A* fell from 0.76 to 0.62. R2-target needed slightly more data: its median was 0.65 at n=50 and 0.63 at n=200, and it matched EW from n=500, with a 95th percentile of 0.65 at n=500–1000 and 0.62 from n=2000. Amplification (A≥1) occurred for either rule only in 1.2% of the draws with n=25 and in a single draw with n=50; from n=100 every draw stayed below 1. Re-estimating reliability on the target population therefore did not improve on equal weights at any calibration size, and its additional parameters made the rule less stable when labeled data were scarce. In this evaluated setting, what a deployment mainly needed was a few hundred labeled target events to place the threshold; the number required depends on the target distribution and on the validators, and is not a general minimum. This applies to threshold placement under a stationary target distribution; it is not an online mechanism that tracks changes in validator reliability over time.

### 4.6. Robustness Under Degraded Validators and Imperfect Human Review

Table 13 describes the degraded twins. Under the transfer rule used for all validators, directed flipping of 80% of the positive training labels left the strongest validators close to their honest versions: the MCC of the refrigerator validator moved from 0.39 to 0.38, that of the motion-light validator from 0.52 to 0.50, and that of the weather-station validator from 0.44 to 0.47. Poisoning changed the alert rates of several weak validators in both directions (GPS tracker from 0.02 to 0.27, garage door from 0.03 to 0.94, thermostat from 0.18 to 0.01). Obsolescence and threshold bias were more damaging: threshold bias reduced every validator’s alert rate to 0.01–0.02 and its MCC to between −0.05 and 0.08, and obsolescence reduced the MCC of the refrigerator validator from 0.39 to −0.03 and that of the motion-light validator from 0.52 to 0.31.

**Figure 9:**
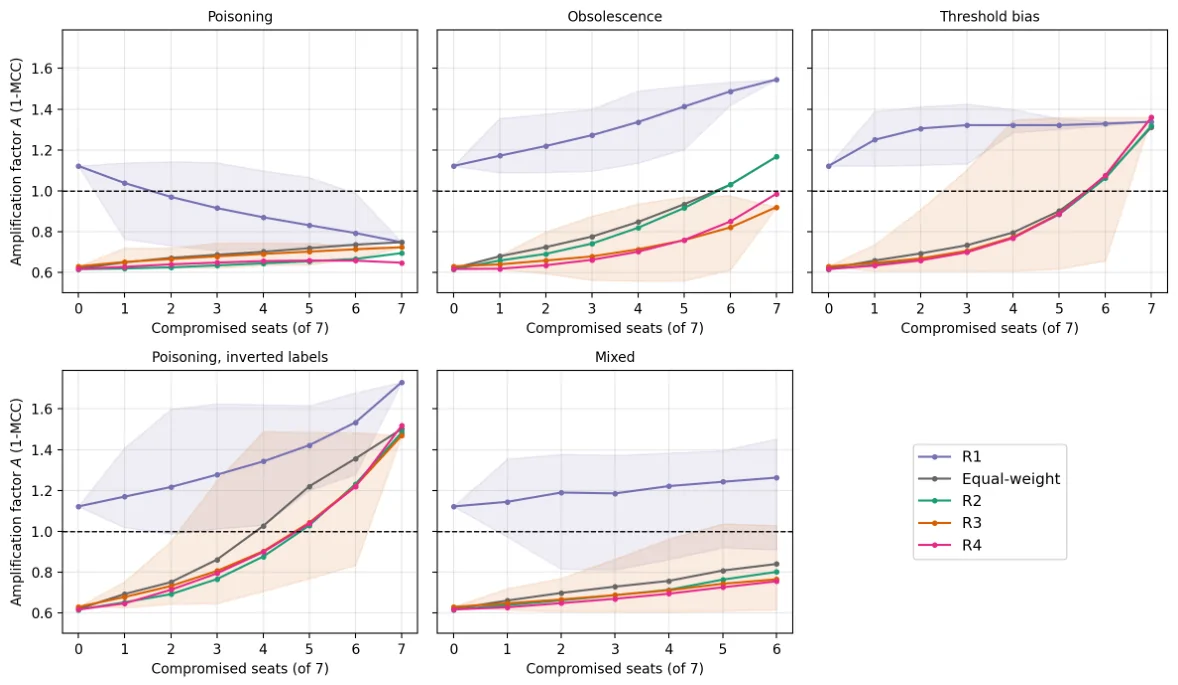
Mean amplification factor (1−MCC) versus the number of compromised seats in a seven-member committee, for the three mechanisms, an extreme label-inversion variant of poisoning, and a mixed scenario. Bands show the minimum–maximum range over subsets for R1 and R3 (5th–95th percentile for the mixed scenario, shown up to c=6). The horizontal reference at A=1 denotes the boundary between error reduction and amplification.

Table 14 and Figure 9 report the amplification factor as the number of compromised seats grows from 0 to 7; with c=0 the values are those of the honest committee. Fixed-threshold majority voting was the most fragile rule under the two mechanisms that degraded validators. Under obsolescence and threshold bias, R1 was above A=1 in every subset for every c≥1 (1.17 and 1.25 at c=1), whereas the mean of every learned-threshold rule stayed below 1 up to c=5. Individual subsets of EW and R2 exceeded A=1 from c=3 under both mechanisms (maximum 1.13 under obsolescence and 1.20 under threshold bias), and the means of EW and R2 crossed 1 at c=6. Under threshold bias the weighted rules behaved like EW and crossed at the same point (1.06–1.08 at c=6), because all biased validators share a near-zero alert rate and similar reliabilities. Under obsolescence, margin weighting retained an advantage in the main setting: R3 stayed below 1 in every subset even with all seven seats degraded (0.92), and the mean of R4 reached only 0.99, although some of its subsets exceeded 1 from c=5. The severity analysis shows that this advantage is specific to that setting. With training frozen after 25 or 100 attacks instead of 50, R3 and R4 crossed A=1 at c=5 or 6, like EW and R2, as Table 15 shows.

Poisoning behaved differently. Under 80% directed flipping, no subset of any learned-threshold rule exceeded A=1 for any *c*, and their mean amplification rose only from 0.62–0.63 to 0.65–0.75 at c=7. The mean amplification of R1 *decreased* as seats were poisoned (from 1.12 to 0.75): the changed alert rates of weak validators happened to move the fixed-threshold majority toward better balanced decisions, an effect that reflects the arbitrariness of a fixed threshold and not a benefit of poisoning. The absence of amplification under poisoning mainly reflects that the simulated attack barely degrades validators under alert-budget transfer, and it should not be read as robustness to stronger poisoning. Severity analysis supports this reading: with 50%, 80%, or 95% of positive labels flipped, or with a 30% symmetric random flip, the mean amplification of the learned-threshold rules at c=3 stayed between 0.61 and 0.69. Only complete label inversion degraded the committee appreciably. Its mean amplification at c=4 was 1.03 for EW and 0.88–0.90 for the weighted rules, and the weighted rules crossed A=1 in expectation at c=5 (1.03–1.04), one seat later than EW. The mixed scenario lay between the individual mechanisms (R3: 0.69 at c=3 and 0.77 at c=6; EW: 0.73 and 0.84; R1: 1.19 and 1.26). The ordering of the two families was stable across all severities: obsolescence after 25, 50, or 100 attacks gave a mean amplification at c=3 of 0.66–0.84 for the learned-threshold rules and 1.27 for R1, and the four threshold percentiles gave 0.69–0.74 and 1.29–1.32. In no variant was the conclusion that fixed-threshold majority voting is more fragile than learned-threshold aggregation reversed. Because each seat is the degraded twin of a distinct source, these results describe the rules under specified degradation profiles; they do not establish how many independent adversarial identities a committee of a given size can tolerate.

**Figure 10:**
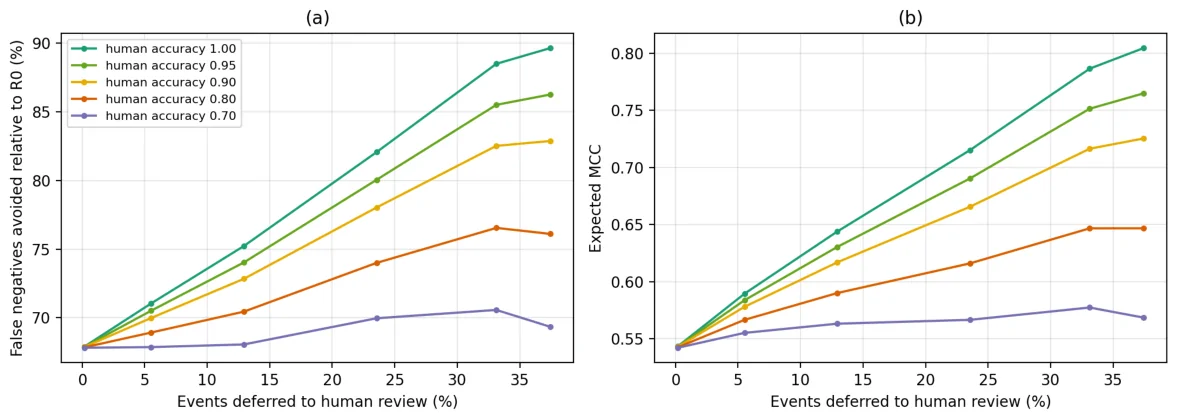
Effect of deferral to human review for R3 as a function of reviewer accuracy: (**a**) false negatives avoided relative to R0 versus the proportion of events deferred; (**b**) expected MCC versus the proportion deferred. Points correspond to δ∈{0,0.03,0.06,0.10,0.15,0.20}.

The second imperfect component is the human reviewer. Deferring events whose R3 score lay within δ of the learned threshold reduced the false negatives left by the committee, but Table 16 and Figure 10 show that the benefit depended on the reviewer’s accuracy. Without deferral, R3 avoided 67.6% of the false negatives of R0 on the evaluation half, with an MCC of 0.542. With δ=0.06 (12.9% of events deferred) and a perfectly accurate reviewer, this rose to 75.2% and the expected MCC to 0.644. With a reviewer of accuracy 0.90, the figures were 72.8% and 0.617, and with accuracy 0.70, 68.1% and 0.563, almost no improvement over not deferring. Wider margins amplified the difference between reviewers: at δ=0.20 (37.4% of events deferred), a perfect reviewer avoided 89.7% of the false negatives, whereas a reviewer of accuracy 0.70 avoided 69.3% and raised MCC only to 0.569, after reviewing more than a third of all events. An idealized perfect reviewer therefore overstates the benefit of deferral, and the deferred region must be sized to measured reviewer accuracy and capacity. Because deferring more than a third of events is unlikely to be compatible with realistic capacity, margins above δ=0.10 should be read as a sensitivity range and not as an operating recommendation.

### 4.7. Complementary Experiment: Federated Operating-Point Failure Under Domain Shift

This experiment is separate from the committee analysis: it uses a different dataset and model, and it illustrates the failure of a shared threshold independently of any verification layer. The model, trained across the ten clients of the hybrid dataset, achieved near-perfect performance on the four Edge-IIoTset clients, with AUC values of 0.997–0.999 and MCC values of 0.968–0.988. In contrast, it obtained an MCC of exactly 0 on both Patients+Alerts clients despite retaining AUC values of 0.937–0.938. Table 17 aggregates this comparison by domain and distinguishes discrimination (AUC), aggregate probabilistic calibration (ECE and Brier score), and decision utility under the shared threshold (MCC).

Discrimination remained high in IoMT (AUC =0.938, compared with 0.993 in IoT), and the aggregate IoMT calibration values (ECE =0.038, Brier score =0.040) did not indicate a large average probability error, although these aggregate measures should not be read as evidence of satisfactory behavior for the minority negative class in a domain with an anomaly prevalence of 0.951. Decision utility at the shared threshold nevertheless collapsed. All 10,001 held-out IoMT traces were classified as positive, including the 490 negative observations, whose minimum predicted probability (0.655) remained above the shared threshold. The model retained the ability to rank IoMT observations but failed to produce both predicted classes at the selected operating point. This is an operating-point failure under domain and prevalence shift rather than a loss of discrimination, and domain-specific threshold adaptation would address it directly, whereas aggregate ECE and Brier values alone do not reveal the loss of negative-class decisions. The relevance to the committee analysis is by analogy: in both cases a single threshold applied across heterogeneous domains, whether the fixed 0.5 of majority voting or the shared threshold of the federated model, fails even though the underlying scores retain ranking information.

**Figure 11:**
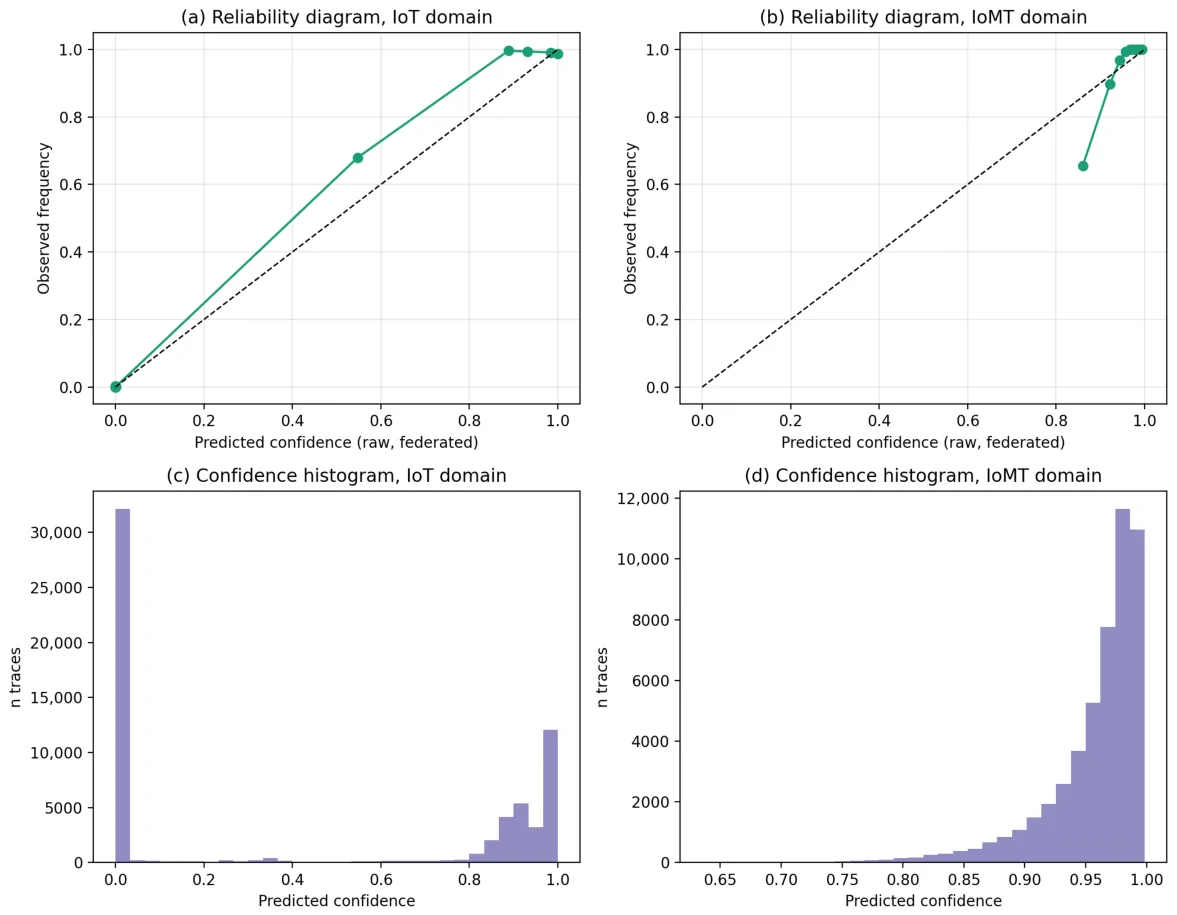
Domain-specific calibration and predicted-probability distributions of the federated detector on the held-out test set: (**a**) reliability diagram for the IoT domain; (**b**) reliability diagram for the IoMT domain; (**c**) predicted-probability histogram for the IoT domain; and (**d**) predicted-probability histogram for the IoMT domain.

In Figure 11a, the IoT reliability curve remains comparatively close to the diagonal at high predicted probabilities and approaches the origin at low predicted probabilities. In Figure 11b, the IoMT calibration bins occupy a compressed high-probability range, approximately 0.86–1.00; this bin-level range does not imply that every individual probability exceeds 0.86. Figure 11c shows a bimodal IoT probability distribution, with concentrations near zero and one, and Figure 11d shows that IoMT probabilities are concentrated predominantly above 0.85, with a lower tail that remains above the shared threshold. One client contained 960 test traces and no observed positive cases, so AUC and MCC could not be estimated for it; the model nevertheless assigned this benign population a mean predicted attack probability of 0.273. A leakage audit conducted before training showed that an aggregated anomalous-event-ratio feature agreed with the trace-level label in 99.99% of the 113,477 records; it encoded the operational definition of the target, was excluded before training, and did not contribute to any reported federated result.

## 5. Discussion

The comparison between fixed-threshold majority voting and learned-threshold score aggregation has a clear answer on this committee, and it concerns the threshold. Majority voting with a threshold of 0.5 produced a point estimate in the direction of amplification (A=1.12 for 1−MCC; 1.34 for the false-negative rate), because several validators rarely vote positive outside their training populations and a quorum of four is seldom reached. Aggregating exactly the same votes into a score and learning the threshold on calibration data reversed the direction (A≈0.62). For the equal-weight score, the learned threshold of 1/7 is a low-quorum rule that alerts when two of seven validators agree, and it transferred unchanged to every held-out device. Reliability weighting, margin weighting, and their hybrid reached the same amplification factor as equal weights, with paired differences whose intervals include zero, and the best single validator was within the same range. A comparison of R2–R4 with R1 alone would attribute to weighting an improvement that is explained by the threshold, which is the confound that the equal-weight baseline was designed to remove.

The correction of the margin definition in this version reinforces that conclusion rather than changing it. Measured from each validator’s own vote boundary, the margin became a weak but correctly oriented indicator of vote correctness, and the margin-weighted rules moved to a higher-precision, lower-recall operating point with the same MCC. Weighting therefore changes where on the precision–recall trade-off the committee operates, but not how well it operates at its best threshold. The setting in which this difference mattered was natural prevalence, where the higher-precision rules retained a larger part of their benefit (A=0.72–0.75 versus 0.82–0.83); this is again an effect of the operating point, and it implies that the threshold should be learned at the prevalence at which it will be used.

The design shares the multi-model structure of an ensemble but differs from one in using population-specific validators, locally estimated reliability, degraded members, and the local detector as the reference decision. It is not a Byzantine fault-tolerant protocol, as it provides no quorum, safety, or liveness guarantees. Separately trained validators were not statistically independent: the median absolute error correlation was 0.19 and the effective number of independent validators was about 4.1 of 7, and the learned-threshold rules were driven by two or three validators with transferable signal.

The calibration-set analysis separates two questions that are often conflated. For the source-domain reliabilities, sampling error was negligible (95% half-widths of at most 0.004–0.011), whereas the transfer gap reached 0.22; collecting more labeled data from a validator’s own population would make its reliability estimate more precise without making it more accurate for the events it is asked to verify. For target-domain estimates, the transfer bias disappears but sampling error dominates at small calibration sizes: the reliability ranking of the seven validators was unstable below a few hundred labeled events and fully reproducible only from about 2000. The decision, however, was insensitive to this instability. Normalized reliability weights move little when individual reliabilities move, and the threshold, which is a single parameter, was recovered from about 500 labeled events in practically every draw of this evaluation. Equal weights with a learned threshold reached their full-calibration performance with fewer labels than reliability weighting, and reliability weighting never improved on them. The practical requirement for learned-threshold aggregation is therefore a modest set of labeled events from the target population, used to place the threshold, rather than precise reliability estimates for each validator.

Several results limit how far the favorable point estimates can be generalized. First, device-level intervals for 1−MCC include the neutral value for every rule, including R1, because only seven devices are available as resampling units. Second, the benefit is not uniform: the committee improved the devices whose local detectors were weak and degraded or did not help those with strong local detectors (refrigerator, motion light). Third, the pooled evaluation uses a high-prevalence sample (0.604 versus natural rates of 0.127–0.224), and reweighting to natural prevalence reduced the estimated reduction in 1−MCC from about 38% to between 17% and 28%, with intervals that reach 1. The leave-one-device-out analysis showed that the equal-weight threshold transfers to an unseen device, whereas the thresholds of the weighted rules were less stable, so the limitation lies in the number of devices and the prevalence regime rather than in overfitting of the simplest rule.

The stress test must be read within its design. Every compromised seat was the degraded twin of a distinct source, so the test does not rely on repeating identities, but it enumerates specific degradation profiles and does not model an adaptive adversary. Under the alert-budget transfer rule, label flipping of up to 95% of positive labels, or a 30% symmetric flip, hardly degraded the validators, which is why no learned-threshold rule amplified error under those variants; this reflects a weak attack under this pipeline and not committee robustness. Obsolescence, threshold bias, and extreme label inversion did degrade the committee and took the learned-threshold rules to or beyond neutrality when most seats were compromised, while fixed-threshold voting amplified error from the first compromised seat. Weighting provided about one additional seat of tolerance under complete label inversion and, in one obsolescence setting, a larger margin that did not persist at other severities. Because the committee size is fixed at seven, the test supports no statement about the committee size needed for a given compromised fraction; a supplementary committee-size grid that sampled three adversarial identities with replacement is reported for exploratory purposes only.

The synthetic layer overstated the amplification of fixed-threshold majority voting even at matched committee size, so conclusions about voting rules drawn from label-conditioned synthetic votes alone should be treated with caution. The federated experiment, which is a separate component, illustrates the same phenomenon at the detector level: a single threshold shared across domains with different base rates collapsed decision utility (MCC =0) in the IoMT domain despite high AUC and low aggregate ECE and Brier score. Aggregate calibration metrics can therefore conceal the loss of minority-class decisions, which is related to, but distinct from, the broader calibration degradation under domain shift described in the literature [11]. Deferral to human review, finally, was valuable only with an accurate reviewer: with an accuracy of 0.70, deferring more than a third of all events yielded almost no improvement.

The study has several limitations. The evaluation asks every validator to score events from all seven devices, including devices whose sensing modality differs from its own training population, for example a refrigerator temperature detector scoring garage-door states. Poor transfer in this design therefore combines non-IID heterogeneity within a common detection task with cross-modality transfer between devices that are not comparable, and the two cannot be separated with the present data; the results describe a cross-checking layer that receives alerts from detectors built on other device types, not a committee of detectors for the same device type. The study evaluates a single verification round and seven IoT populations from one data-collection campaign (TON_IoT), whose telemetry predates more recent IoT benchmarks and may not represent current devices, protocols, or attack distributions. The pool is dominated by attacks relative to natural rates. Calendar variables were removed because they leaked attack timing, but burst-rate and interarrival features may still partly reflect campaign synchronization. Validator errors are correlated, and none of the rules model this dependence. The reference decision R0 assumes that the local detector of the originating device is the appropriate comparator. The decision margin is only weakly associated with vote correctness, and for a large share of votes it is zero because isotonic recalibration places them on the plateau that contains the vote boundary. The stress test relies on three simulated degradation mechanisms with distinct but non-adaptive profiles, and the human-review analysis uses a symmetric, accuracy-parameterized reviewer model that does not capture workload effects. Finally, the host and network validators evaluated separately transferred little information to the IoT pool and are excluded from the main analysis.

Three implications follow, with the hedging that the evidence requires. First, a fixed-threshold majority vote across heterogeneous detectors is a risky default; aggregating votes into a score and learning its threshold on labeled calibration data is a more defensible baseline, and an equal-weight version of that score should be the reference against which any weighting scheme is judged. Second, the threshold should be learned on labeled events from the target population and at its prevalence; a few hundred such events sufficed in this setting, although the number needed depends on the target distribution and on the validators, whereas reliability estimates from the validators’ source populations were biased in ways that more source data cannot correct. Third, the decision to replace a local detector by the committee should be assessed per device, because the committee helps weak local detectors and can hurt strong ones, and any deployment should monitor per-validator alert rates and error dependence and be validated under new device populations and natural prevalence.

## 6. Conclusions

This study compared fixed-threshold majority voting with learned-threshold score aggregation for verifying IoT security events with seven separately trained detectors and 56,000 shared events, using the detector local to each event as the reference. Fixed-threshold majority voting produced point estimates in the direction of amplification (A=1.12 for 1−MCC; 1.34 for the false-negative rate), although device-level intervals included A=1. Aggregating the same votes into a score with a threshold learned on calibration data reversed the direction (A≈0.62). An equal-weight score with a learned threshold matched reliability-, margin-, and hybrid-weighted scores and transferred unchanged to held-out devices, so the difference between the two families of rules is explained by threshold placement rather than by weighting. With the margin measured from each validator’s own vote boundary, margin weighting shifted the committee toward higher precision at the same MCC, which helped under natural prevalence but did not improve the committee at its best threshold.

The calibration data required by learned-threshold aggregation were modest in this evaluation: about 500 labeled target events placed the threshold in practically every draw, whereas the reliabilities needed for weighting were either biased (source domain, by up to 0.22 despite sampling half-widths of at most 0.011) or unstable in their ranking (target domain, below a few thousand events). The benefit of aggregation was heterogeneous across devices and shrank under natural prevalence. Under degraded validators, fixed-threshold majority voting amplified error from the first seat compromised by obsolescence or threshold bias, whereas the learned-threshold rules remained below neutrality on average with up to five of seven seats degraded; poisoning barely affected the validators, and weighting added about one seat of tolerance only under complete label inversion. Deferral to human review helped only with accurate reviewers. A synthetic vote layer overstated the amplification of majority voting even at matched committee size, and a complementary federated experiment showed that a single shared threshold can collapse decision utility (MCC =0) in a domain with a different base rate despite an AUC of 0.94.

These findings are limited by the use of seven device populations from one collection campaign, a high-prevalence evaluation pool, correlated validator errors, and simulated degradation profiles that do not model adaptive adversaries. Future work should replicate the comparison with more recent IoT benchmarks and many more devices, learn thresholds online under changing prevalence, model error dependence explicitly, evaluate adaptive adversaries and committee-size trade-offs with independently built adversarial validators, and test deferral policies with measured reviewer accuracy.

## Figures and Tables

**Table 1:** Population size, natural anomaly rate, and attack diversity of the seven IoT device populations used to train the committee validators.

Device	Records Used	Natural Anomaly Rate	Attack Types
Refrigerator	587,076	0.147	7
GPS tracker	595,686	0.137	8
Garage door	570,111 ^a^	0.129	8
Modbus channel	287,194	0.224	6
Motion light	452,262	0.141	8
Thermostat	437,607 ^b^	0.127	7
Weather station	650,242	0.139	8

^a^ A total of 21,335 records with unparsable timestamps were removed. ^b^ A total of 4621 records with unparsable timestamps were removed.

**Table 2:** Primary signal selected for each IoT device population and its semantic type.

Population	Primary Signal	Type
Refrigerator	fridge_temperature	Sensor reading
GPS tracker	latitude	Sensor reading
Garage door	door_state	Categorical, ordinally encoded
Modbus channel	FC1_Read_Input_Register	Sensor/actuator reading
Motion light	motion_status	Sensor reading
Thermostat	current_temperature	Sensor reading
Weather station	temperature	Sensor reading

**Table 3:** Individual performance of the seven validators on the evaluation half of the shared pool, and the share of total vote weight that each validator receives under equal weighting and under rules R2–R4 (mean over events). Reliability $\rho_{j}$ is the balanced accuracy on the validator’s own calibration segment.

Validator	$\rho_{j}$	AUC	MCC	Alert Rate	Equal (%)	R2 (%)	R3 (%)	R4 (%)
Fridge	0.91	0.761	0.386	0.600	14.3	19.0	17.0	20.1
GPS Tracker	0.53	0.511	0.022	0.022	14.3	11.1	0.0	0.0
Garage Door	0.52	0.606	−0.041	0.030	14.3	10.9	14.6	11.0
Modbus	0.73	0.642	0.109	0.348	14.3	15.2	29.4	28.1
Motion Light	0.84	0.822	0.523	0.633	14.3	17.5	31.5	34.1
Thermostat	0.57	0.656	0.026	0.184	14.3	11.9	1.4	1.1
Weather	0.69	0.767	0.437	0.680	14.3	14.4	6.1	5.6

**Table 4:** Unweighted majority voting with a fixed threshold (R1) versus the local-detector reference (R0) on 56,000 events. Intervals for 1−MCC and the false-negative rate use per-event bootstrap resampling stratified by device; the Brier-score interval uses a normal approximation. Device-level intervals are reported in the text.Values above A=1 indicate amplification.

Error Measure	Err (R0)	Err (R1)	Amplification Factor	Event-Level 95% CI	Direction
1−MCC	0.729	0.815	1.118	[1.106, 1.130]	Amplification
False-negative rate	0.517	0.688	1.331	[1.319, 1.344]	Amplification
Brier score	0.294	0.231	0.785	[0.778, 0.792]	Error reduction

**Table 5:** Amplification factor of fixed-threshold majority voting for the synthetic layer at three committee configurations and for the real-detector committee. The synthetic reference decision R0 is a single threshold on the generating score, learned on one half of the synthetic events and evaluated on the other half.

Configuration	*A* for 1−MCC	*A* for FNR
Synthetic, all 21 validators	1.23	3.44
Synthetic, 7 random validators (median [2.5–97.5%])	1.28 [1.24, 1.35]	3.29 [3.16, 3.40]
Synthetic, 7 validators with reliability matched to the real committee	1.31	3.25
Real IoT committee (7 trained detectors)	1.12	1.34

**Table 6:** Performance of the local detector, fixed-threshold majority voting, the equal-weight and best-single-validator baselines, and the weighted rules on the held-out evaluation half (n=28,000). *A* is the amplification factor for 1−MCC and for the false-negative rate (FNR); intervals are device-level block-bootstrap 95% intervals (10,000 replicates, 7 devices).

Rule	MCC	Recall	Precision	FNR	$A_{\mathrm{MCC}}$	95% CI	$A_{\mathrm{FNR}}$	95% CI
R0 (local detector)	0.272	0.483	0.775	0.517	1.00	–	1.00	–
R1 (majority, fixed 0.5)	0.184	0.309	0.761	0.691	1.12	[0.84, 1.69]	1.34	[0.83, 3.63]
Equal-weight mean vote, learned threshold	0.549	0.942	0.759	0.058	0.62	[0.31, 1.10]	0.11	[0.05, 0.27]
Best single validator (Motion Light)	0.523	0.837	0.798	0.163	0.66	[0.45, 1.06]	0.31	[0.19, 0.84]
R2 (static reliability)	0.552	0.949	0.756	0.051	0.62	[0.31, 1.10]	0.10	[0.05, 0.23]
R3 (structural margin)	0.542	0.833	0.811	0.167	0.63	[0.35, 1.09]	0.32	[0.15, 1.07]
R4 (hybrid)	0.551	0.828	0.819	0.172	0.62	[0.34, 1.09]	0.33	[0.16, 1.06]

**Table 7:** MCC on the evaluation half after removing one validator at a time (thresholds re-learned on the calibration half for every committee). The MCC of R0 is 0.272.

Validator Removed	R1	EW	R2	R3	R4
None (full committee)	0.184	0.549	0.552	0.542	0.551
Fridge	0.031	0.498	0.521	0.481	0.522
GPS Tracker	0.171	0.551	0.553	0.542	0.551
Garage Door	0.195	0.551	0.552	0.544	0.558
Modbus	0.055	0.542	0.549	0.559	0.559
Motion Light	−0.030	0.477	0.501	0.463	0.463
Thermostat	0.187	0.523	0.541	0.550	0.560
Weather	0.008	0.506	0.540	0.544	0.562

**Table 8:** MCC within each device on the evaluation half of the shared pool for the local detector (R0), fixed-threshold majority voting (R1), the equal-weight baseline (EW), the best single validator (BV), and rules R2–R4.

Device	Anomaly Rate	R0	R1	EW	BV	R2	R3	R4
Fridge	0.586	0.912	0.430	0.829	0.642	0.830	0.797	0.808
GPS Tracker	0.655	−0.032	0.129	0.828	0.623	0.820	0.758	0.746
Garage Door	0.663	0.138	0.343	0.678	0.608	0.686	0.688	0.684
Modbus	0.400	0.045	−0.062	−0.031	0.192	−0.026	0.105	0.111
Motion Light	0.601	0.500	0.111	0.070	0.500	0.051	0.184	0.152
Thermostat	0.654	0.070	0.390	0.818	0.716	0.816	0.826	0.816
Weather	0.664	0.673	0.300	0.694	0.532	0.700	0.616	0.715
Devices with MCC above R0	–	–	3	4	4	4	4	5

**Table 9:** MCC and amplification factor (1−MCC, device-level 95% interval) under three weightings of the evaluation events: the pool as evaluated, natural device sizes, and natural device sizes together with natural anomaly rates.

Rule	Pool as Evaluated			Natural Size			Natural Size and Prevalence		
	MCC	A	95% CI	MCC	A	95% CI	MCC	A	95% CI
R0 (local detector)	0.272	–	–	0.310	–	–	0.299	–	–
R1 (majority, fixed 0.5)	0.184	1.12	[0.84, 1.69]	0.225	1.12	[0.84, 1.80]	0.251	1.07	[0.85, 1.32]
Equal-weight mean vote, learned threshold	0.549	0.62	[0.31, 1.10]	0.611	0.56	[0.31, 1.05]	0.424	0.82	[0.58, 1.10]
Best single validator (Motion Light)	0.523	0.66	[0.45, 1.06]	0.553	0.65	[0.45, 1.12]	0.427	0.82	[0.62, 1.09]
R2 (static reliability)	0.552	0.62	[0.31, 1.10]	0.613	0.56	[0.31, 1.05]	0.419	0.83	[0.59, 1.11]
R3 (structural margin)	0.542	0.63	[0.35, 1.09]	0.594	0.59	[0.34, 1.07]	0.474	0.75	[0.51, 1.02]
R4 (hybrid)	0.551	0.62	[0.34, 1.09]	0.607	0.57	[0.34, 1.06]	0.497	0.72	[0.49, 0.99]

**Table 10:** MCC on the evaluation half of each device when the aggregation threshold is learned on the calibration halves of the other six devices.

Held-Out Device	R0	EW	R2	R3	R4
Fridge	0.912	0.829	0.830	0.793	0.804
GPS Tracker	−0.032	0.828	0.820	0.235	0.746
Garage Door	0.138	0.678	0.678	0.653	0.684
Modbus	0.045	−0.031	−0.026	0.105	0.117
Motion Light	0.500	0.070	−0.159	0.175	0.150
Thermostat	0.070	0.818	0.816	0.826	0.814
Weather	0.673	0.694	0.645	0.616	0.635
Pooled (device-balanced)	0.272	0.549	0.509	0.471	0.541

**Table 11:** Source-domain reliability estimates: size of each validator’s calibration segment, number of attacks in it, estimated reliability ${\hat{\rho }}_{j}$, oracle reliability on the pool, upper bound of the 95% sampling half-width, and transfer gap (estimate minus oracle).

Validator	$n_{j}$	$n_{j}^{+}$	${\hat{\rho }}_{j}$	Oracle $\rho_{j}$	Max. 95% Half-Width	Transfer Gap
Fridge	20,944	12,225	0.91	0.69	0.0069	0.22
GPS Tracker	52,391	19,825	0.53	0.50	0.0044	0.03
Garage Door	42,515	12,623	0.52	0.49	0.0052	0.03
Modbus	33,013	12,958	0.73	0.55	0.0055	0.18
Motion Light	15,773	9663	0.84	0.76	0.0080	0.08
Thermostat	21,208	2466	0.57	0.51	0.0105	0.06
Weather	74,061	20,233	0.69	0.71	0.0040	−0.02

**Table 12:** Target-domain calibration with *n* labeled events drawn from the calibration half of the pool (1000 draws per *n*): mean and maximum over validators of the standard deviation of ${\hat{\rho }}_{j}$; median Kendall τ between the reliability ranking and the full-half ranking; median (95th percentile) of the largest deviation of an R2 weight share from its full-half value, in percentage points; and median (95th percentile) of the amplification factor (1−MCC, evaluation half) of EW and of R2 with target reliabilities.

*n*	Mean SD $\hat{\rho }$	Max SD $\hat{\rho }$	Kendall τ	Weight Dev. (pp)	EW *A*	R2-Target *A*
25	0.076	0.102	0.62	2.73 [4.83]	0.62 [0.76]	0.66 [0.82]
50	0.052	0.068	0.71	1.82 [3.23]	0.62 [0.76]	0.65 [0.75]
100	0.037	0.047	0.81	1.25 [2.33]	0.62 [0.76]	0.64 [0.74]
200	0.026	0.034	0.81	0.94 [1.64]	0.62 [0.73]	0.63 [0.71]
500	0.016	0.021	0.90	0.56 [0.99]	0.62 [0.62]	0.62 [0.65]
1000	0.011	0.015	0.90	0.39 [0.68]	0.62 [0.62]	0.62 [0.65]
2000	0.008	0.010	1.00	0.28 [0.48]	0.62 [0.62]	0.62 [0.62]
5000	0.005	0.006	1.00	0.16 [0.28]	0.62 [0.62]	0.62 [0.62]
14,000	0.002	0.003	1.00	0.08 [0.14]	0.62 [0.62]	0.62 [0.62]

**Table 13:** MCC and alert rate of each validator on the shared pool in its honest version and in the degraded version of the same source under each mechanism: poisoning (80% of positive labels flipped), obsolescence (training frozen after 50 attack samples), and threshold bias (98th percentile of calibration scores).

Source	Honest		Poisoned		Obsolete		Threshold-Biased	
	MCC	Alert	MCC	Alert	MCC	Alert	MCC	Alert
Fridge	0.39	0.60	0.38	0.60	−0.03	0.72	0.05	0.02
GPS Tracker	0.03	0.02	0.01	0.27	−0.09	0.01	0.01	0.01
Garage Door	−0.04	0.03	−0.05	0.94	−0.01	0.01	−0.05	0.02
Modbus	0.11	0.35	0.20	0.35	−0.11	0.05	−0.04	0.02
Motion Light	0.52	0.63	0.50	0.61	0.31	0.82	0.06	0.02
Thermostat	0.02	0.18	−0.05	0.01	−0.06	0.07	−0.03	0.02
Weather	0.44	0.68	0.47	0.80	−0.00	0.01	0.08	0.02

**Table 14:** Amplification factor (1−MCC) as a function of the number of compromised seats *c* in a seven-member committee: mean over all 7c subsets and, in brackets, the minimum and maximum over subsets. Mixed rows average 300 random assignments of mechanisms to compromised seats, and brackets give the 5th and 95th percentiles. Values above 1 indicate amplification relative to the honest local detector.

Mechanism	*c*	R1	EW	R2	R3	R4
poisoning	0	1.12 [1.12, 1.12]	0.62 [0.62, 0.62]	0.62 [0.62, 0.62]	0.63 [0.63, 0.63]	0.62 [0.62, 0.62]
	1	1.04 [0.76, 1.14]	0.65 [0.61, 0.70]	0.62 [0.60, 0.64]	0.65 [0.62, 0.72]	0.63 [0.60, 0.67]
	2	0.97 [0.73, 1.14]	0.67 [0.60, 0.73]	0.62 [0.58, 0.66]	0.67 [0.62, 0.72]	0.64 [0.60, 0.68]
	3	0.92 [0.71, 1.14]	0.69 [0.61, 0.75]	0.63 [0.59, 0.69]	0.68 [0.62, 0.74]	0.65 [0.60, 0.68]
	4	0.87 [0.72, 1.10]	0.70 [0.61, 0.76]	0.64 [0.59, 0.70]	0.69 [0.63, 0.75]	0.66 [0.62, 0.69]
	7	0.75 [0.75, 0.75]	0.75 [0.75, 0.75]	0.69 [0.69, 0.69]	0.72 [0.72, 0.72]	0.65 [0.65, 0.65]
obsolescence	0	1.12 [1.12, 1.12]	0.62 [0.62, 0.62]	0.62 [0.62, 0.62]	0.63 [0.63, 0.63]	0.62 [0.62, 0.62]
	1	1.17 [1.09, 1.35]	0.68 [0.62, 0.79]	0.66 [0.62, 0.78]	0.64 [0.61, 0.68]	0.62 [0.61, 0.65]
	2	1.22 [1.09, 1.38]	0.72 [0.63, 0.93]	0.69 [0.62, 0.88]	0.66 [0.59, 0.80]	0.64 [0.58, 0.79]
	3	1.27 [1.10, 1.40]	0.78 [0.63, 1.13]	0.74 [0.62, 1.13]	0.68 [0.56, 0.88]	0.66 [0.56, 0.88]
	4	1.34 [1.14, 1.49]	0.85 [0.63, 1.16]	0.82 [0.63, 1.16]	0.71 [0.56, 0.94]	0.70 [0.56, 0.98]
	7	1.55 [1.55, 1.55]	1.17 [1.17, 1.17]	1.17 [1.17, 1.17]	0.92 [0.92, 0.92]	0.99 [0.99, 0.99]
threshold bias	0	1.12 [1.12, 1.12]	0.62 [0.62, 0.62]	0.62 [0.62, 0.62]	0.63 [0.63, 0.63]	0.62 [0.62, 0.62]
	1	1.25 [1.12, 1.39]	0.66 [0.62, 0.72]	0.64 [0.62, 0.69]	0.65 [0.61, 0.74]	0.63 [0.61, 0.74]
	2	1.31 [1.13, 1.41]	0.69 [0.62, 0.81]	0.66 [0.62, 0.81]	0.67 [0.61, 0.91]	0.66 [0.61, 0.90]
	3	1.32 [1.13, 1.42]	0.73 [0.63, 1.20]	0.70 [0.62, 1.20]	0.71 [0.61, 1.10]	0.70 [0.61, 1.10]
	4	1.32 [1.28, 1.40]	0.80 [0.63, 1.30]	0.77 [0.63, 1.30]	0.77 [0.61, 1.35]	0.77 [0.61, 1.35]
	7	1.34 [1.34, 1.34]	1.31 [1.31, 1.31]	1.32 [1.32, 1.32]	1.36 [1.36, 1.36]	1.36 [1.36, 1.36]
poisoning, inverted labels	1	1.17 [1.02, 1.41]	0.69 [0.63, 0.76]	0.65 [0.61, 0.72]	0.68 [0.63, 0.75]	0.65 [0.60, 0.74]
	2	1.22 [0.99, 1.60]	0.75 [0.63, 0.84]	0.69 [0.62, 0.81]	0.73 [0.64, 0.95]	0.71 [0.63, 0.95]
	3	1.28 [1.01, 1.62]	0.86 [0.72, 1.24]	0.77 [0.64, 1.21]	0.81 [0.65, 1.25]	0.79 [0.64, 1.24]
	4	1.34 [1.03, 1.62]	1.03 [0.77, 1.37]	0.88 [0.67, 1.34]	0.90 [0.71, 1.49]	0.90 [0.70, 1.49]
	7	1.73 [1.73, 1.73]	1.50 [1.50, 1.50]	1.48 [1.48, 1.48]	1.47 [1.47, 1.47]	1.52 [1.52, 1.52]
mixed	1	1.14 [0.97, 1.35]	0.66 [0.61, 0.79]	0.64 [0.60, 0.78]	0.65 [0.61, 0.72]	0.63 [0.61, 0.67]
	2	1.19 [0.82, 1.38]	0.70 [0.62, 0.80]	0.66 [0.60, 0.78]	0.67 [0.61, 0.77]	0.65 [0.60, 0.77]
	3	1.19 [0.81, 1.37]	0.73 [0.63, 0.88]	0.69 [0.60, 0.85]	0.69 [0.61, 0.86]	0.67 [0.59, 0.88]
	4	1.22 [0.86, 1.38]	0.76 [0.63, 0.94]	0.71 [0.60, 0.89]	0.71 [0.61, 0.96]	0.69 [0.60, 0.95]
	6	1.26 [0.91, 1.45]	0.84 [0.66, 1.16]	0.80 [0.61, 1.16]	0.77 [0.62, 1.03]	0.76 [0.61, 1.03]

**Table 15:** Sensitivity of the stress test to the severity of each mechanism: mean amplification factor (1−MCC) with three compromised seats (c=3) for R1, EW, R2, R3, and R4, and with all seven seats compromised (c=7) for EW and R3. Main settings are marked.

Variant	c=3					c=7	
	R1	EW	R2	R3	R4	EW	R3
Poisoning, 50% of positives flipped	0.93	0.68	0.62	0.64	0.62	0.75	0.63
Poisoning, 80% of positives flipped (main)	0.92	0.69	0.63	0.68	0.65	0.75	0.72
Poisoning, 95% of positives flipped	0.99	0.68	0.63	0.65	0.64	0.77	0.67
Poisoning, 30% symmetric random flip	1.08	0.64	0.61	0.64	0.63	0.70	0.66
Poisoning, all labels inverted (extreme)	1.28	0.86	0.77	0.81	0.79	1.50	1.47
Obsolescence, frozen after 25 attacks	1.27	0.84	0.76	0.76	0.75	1.37	1.37
Obsolescence, frozen after 50 attacks (main)	1.27	0.78	0.74	0.68	0.66	1.17	0.92
Obsolescence, frozen after 100 attacks	1.27	0.82	0.77	0.79	0.78	1.36	1.36
Threshold bias, 90th percentile	1.29	0.74	0.69	0.70	0.69	1.16	1.21
Threshold bias, 95th percentile	1.32	0.73	0.69	0.70	0.70	1.29	1.22
Threshold bias, 98th percentile (main)	1.32	0.73	0.70	0.71	0.70	1.31	1.36
Threshold bias, 99th percentile	1.32	0.73	0.70	0.71	0.70	1.31	1.34

**Table 16:** Effect of the deferral margin δ and the reviewer accuracy *a* on R3 (evaluation half). Columns give the share of events deferred, the share of the false negatives of R0 that are avoided, and the expected MCC. Without deferral, 67.6% of the false negatives are avoided and MCC is 0.542.

δ	*a*	Deferred (%)	FN Avoided (%)	Expected MCC
0.06	1.00	12.9	75.2	0.644
0.06	0.95	12.9	74.0	0.630
0.06	0.90	12.9	72.8	0.617
0.06	0.80	12.9	70.4	0.590
0.06	0.70	12.9	68.1	0.563
0.10	1.00	23.5	82.1	0.715
0.10	0.95	23.5	80.1	0.690
0.10	0.90	23.5	78.0	0.666
0.10	0.80	23.5	74.0	0.616
0.10	0.70	23.5	70.0	0.567

**Table 17:** Domain-level performance of the federated detector under a single shared decision threshold. Results comprise four Edge-IIoTset clients in the IoT domain and two Patients+Alerts clients in the IoMT domain.

Domain	*n* (Test)	Anomaly Rate	AUC	MCC	ECE	Brier Score
IoT (4 clients)	12,697	0.465	0.993	0.965	0.035	0.018
IoMT (2 clients)	10,001	0.951	0.938	0.000	0.038	0.040

## Data Availability

The publicly available datasets analyzed in this study include TON_IoT (UNSW Research, https://research.unsw.edu.au/projects/toniot-datasets, accessed on 16 October 2026) and Edge-IIoTset (IEEE DataPort, https://doi.org/10.21227/mbc1-1h68. The Patients+Alerts component and the synthetic verification layer contain synthetically generated data. The analysis scripts, fixed random seeds, parameter manifests, result files, and the code that generates every table and figure are provided as Supplementary Material (archive “Supplementary_Code_Data”). The shared event pool and the per-validator votes are derived from the public datasets and can be regenerated with the provided scripts; they are available from the corresponding author upon reasonable request. The original third-party datasets are not redistributed and remain subject to the conditions established by their respective providers.

## Supplementary Materials

The following supporting information can be downloaded at https://www.mdpi.com/article/10.3390/s26216969/s1, Supplementary Material (host and network validators, supplementary committee-size grid, additional figures; file Supplementary_Material.pdf) and the code archive (file Supplementary_Code_Data.zip).

## References

[1] Fei W., Ohno H., Sampalli S. (2023). A Systematic Review of IoT Security: Research Potential, Challenges, and Future Directions. ACM Comput. Surv..

[2] Seo J., Catak F.O., Rong C. Understanding Federated Learning from IID to Non-IID dataset: An Experimental Study. Proceedings of the 36th Norsk IKT-Konferanse for Forskning og Utdanning (NIKT).

[3] Buyuktanir B., Altinkaya Ş., Karatas Baydogmus G., Yildiz K. (2025). Federated learning in intrusion detection: Advancements, applications, and future directions. Clust. Comput..

[4] Lamport L., Shostak R., Pease M. (1982). The Byzantine Generals Problem. ACM Trans. Program. Lang. Syst..

[5] Zhang G., Pan F., Mao Y., Tijanic S., Dang’ana M., Motepalli S., Zhang S., Jacobsen H.-A. (2024). Reaching Consensus in the Byzantine Empire: A Comprehensive Review of BFT Consensus Algorithms. ACM Comput. Surv..

[6] Zhang M.-L., Wang D.-B. (2026). Uncertainty Calibration in Deep Learning: Methods, Emerging Challenges, and LLM Frontiers. J. Comput. Sci. Technol..

[7] Goldschmidt P., Chudá D. (2025). Network intrusion datasets: A survey, limitations, and recommendations. Comput. Secur..

[8] Zhong C., Sarkar A., Manna S., Khan M.Z., Noorwali A., Das A., Chakraborty K. (2024). Federated learning-guided intrusion detection and neural key exchange for safeguarding patient data on the internet of medical things. Int. J. Mach. Learn. Cybern..

[9] Beshah Y.K., Abebe S.L., Melaku H.M. (2025). Dynamic weight clustered federated learning for IoT DDoS attack detection. Sci. Rep..

[10] Peng H., Yu H., Tang X., Li X., Salakhutdinov R., Kolter Z., Heller K., Weller A., Oliver N., Scarlett J., Berkenkamp F. (2024). FedCal: Achieving Local and Global Calibration in Federated Learning via Aggregated Parameterized Scaler. Proceedings of the 41st International Conference on Machine Learning, Vienna, Austria, 21–27 July 2024.

[11] Dong J., Jiang Z., Yang B., Zhang T., Dudik M., Jaggi M., Agarwal A., Li S., Schuurmans D., Zhu J., Berkenkamp F., Dong H., Bietti A. (2026). Expectation Consistency Loss: Rethink Confidence Calibration under Covariate Shift. Proceedings of the 43rd International Conference on Machine Learning, Seoul, South Korea, 6–11 July 2026.

[12] Ragab M., Alshammari S.M., Al-Malaise Al-Ghamdi A.S. (2023). Modified Metaheuristics with Weighted Majority Voting Ensemble Deep Learning Model for Intrusion Detection System. Comput. Syst. Sci. Eng..

[13] Wang C., Zhou Z., Zheng G. (2024). Efficient weighted multi-source trust aggregation scheme for edge computing offloading. Soc. Netw. Anal. Min..

[14] Lavaur L., Busnel Y., Autrel F. Systematic Analysis of Label-flipping Attacks against Federated Learning in Collaborative Intrusion Detection Systems. Proceedings of the 19th International Conference on Availability, Reliability and Security.

[15] Yang S., Zheng X., Li J., Xu J., Wang X., Ngai E.C.H. ReCDA: Concept Drift Adaptation with Representation Enhancement for Network Intrusion Detection. Proceedings of the 30th ACM SIGKDD Conference on Knowledge Discovery and Data Mining.

[16] Vassilev A., Oprea A., Fordyce A., Anderson H., Davies X., Hamin M. (2025). Adversarial Machine Learning: A Taxonomy and Terminology of Attacks and Mitigations.

[17] Wei Z., Cao Y., Feng L., Salakhutdinov R., Kolter Z., Heller K., Weller A., Oliver N., Scarlett J., Berkenkamp F. (2024). Exploiting Human-AI Dependence for Learning to Defer. Proceedings of the 41st International Conference on Machine Learning, Vienna, Austria, 21–27 July 2024.

[18] McMahan H.B., Moore E., Ramage D., Hampson S., Agüera y Arcas B., Singh A., Zhu J. (2017). Communication-Efficient Learning of Deep Networks from Decentralized Data. Proceedings of the 20th International Conference on Artificial Intelligence and Statistics, Fort Lauderdale, FL, USA, 20–22 April 2017.

