# Supplementary material for: Majority Voting Versus Learned-Threshold Score Aggregation for Verifying IoT Security Events Under Non-IID Heterogeneity
Source: Sensors (Basel). 2026 Nov 4;26(21):6969. doi: 10.3390/s26216969 (PMC13766526; doi:10.3390/s26216969)
Supplement: Supplementary file 1 [file sensors-26-06969-s001.zip › Supplementary_Material.pdf]

Supplementary Material for "Majority Voting versus Learned-Threshold Score Aggregation for Verifying IoT Security Events under Non-IID Heterogeneity"

This document collects analyses that were obtained with an extended committee of twelve validators and are not used in the main text: the five host and network validators, the committee-size grid with repeated adversarial identities, and the associated figures. They are retained for transparency. They must not be used to derive committee-size recommendations, for the reasons stated in Section 3.

1 Host and Network Validators

Five populations in addition to the seven IoT devices were verified as distinct captures: a process-accounting capture from a Linux host (1,000,000 records; 12.1% anomalous), a disk-I/O capture from a Linux host (1,000,000 records; 19.4% anomalous), a Windows 10 host (35,975 records; 30.9% anomalous), a Windows 7 host (28,367 records; 21.1% anomalous), and one of 23 network-flow capture fragments (1,000,000 records; 79.1% anomalous). A Linux memory capture was excluded because it shared all its timestamps with the process-accounting capture, and three further captures with numerical suffixes were excluded because their separation from the retained captures could not be verified. Table S1 lists the primary signal that the deterministic selection rule chose for each of these populations.

Table S1: Primary signal selected for the host and network populations.

Population	Primary signal	Type
`linux_disk_1`	`PID`	Administrative identifier
Windows 7	`Processor(_Total) DPC Rate`	Performance counter
`Network_1`	`src_port`	Administrative/weak identifier
`Linux_process_1`	`PID`	Administrative identifier
Windows 10	`Processor_DPC_Rate`	Performance counter

The selection rule chose process identifiers for the two Linux populations and a source port for the network-flow population. These variables are not direct behavioral measurements, and a source-specific semantic selection would be preferable for schemas with more than four columns. This limitation does not by itself explain the near-random transfer of the five validators: the Windows 7 and Windows 10 validators used genuine performance counters and also transferred poorly (pooled AUC 0.45 and 0.51, compared with 0.87 and 0.39 in their source domains). Pooled AUC for the five validators on the IoT pool ranged from 0.44 to 0.52, which is consistent with a mismatch between host/network telemetry and IoT sensor behavior. For this reason they were removed from the primary analysis.

2 Error Correlation among the Twelve Validators

Across the 66 pairs of the twelve-validator committee, the median absolute error correlation was 0.21, the mean absolute correlation was 0.29, and 13 pairs had an absolute correlation above 0.5 (Figure S1). Excluding the three validators with administrative primary signals (`linux_disk_1`, `Linux_process_1`, `Network_1`) raised the median absolute correlation of the remaining nine validators to 0.25, because the strongest pairs did not involve them. The seven-validator matrix used in the main text is Figure 3 of the article.

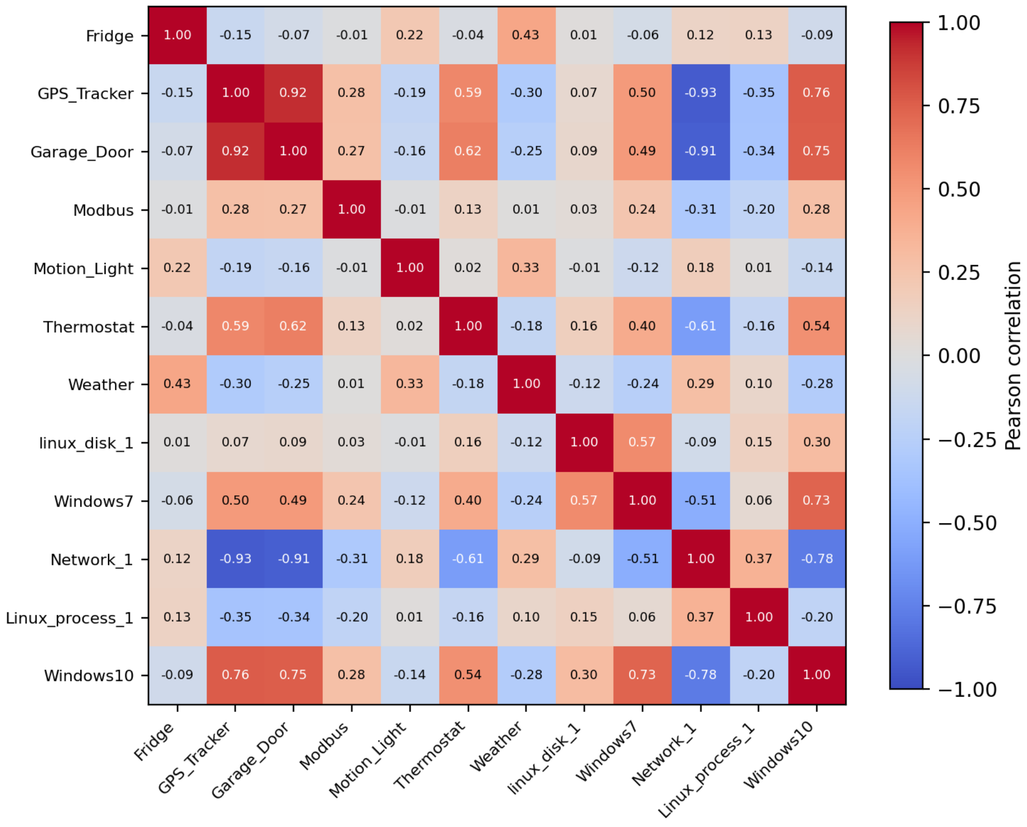

Figure S1: Pairwise correlation of errors among the twelve validators of the extended committee on the shared event pool.

3 Committee-Size Grid with Repeated Adversarial Identities

A supplementary robustness analysis degraded nine of the twelve validators (three per mechanism; poisoning: refrigerator, Modbus channel, motion light; obsolescence: GPS tracker, garage door, weather station; threshold bias: thermostat, Linux process host, Windows 10 host) and evaluated committee sizes $k \in \{3, 5, 7, 9, 11, 12\}$ with compromised-seat fractions $f \in \{0, 0.10, \ldots, 0.50\}$. Because each mechanism had only three identities, compromised seats beyond three were filled by sampling those identities with replacement; a twelve-member committee at 50% compromise, for example, contained six compromised seats made of three distinct identities. Thirty random committees were drawn per cell. Rules R3 and R4 use the decision margin measured from each validator's own vote boundary (Equation 4 of the article), and each degraded twin carries its own boundary.

Table S2 reports the smallest tested compromised fraction at which the median amplification factor exceeded one.

Table S2: Supplementary grid: minimum compromised fraction at which the median amplification exceeded $A = 1$ ("Baseline unstable": already above one before compromise; dash: no crossover up to 50%).

Mechanism	Rule	$k = 3$	$k = 5$	$k = 7$	$k = 9$
Poisoning	R2/R3	Baseline unstable	–	–	–
Poisoning	R4 (hybrid)	–	–	–	–
Threshold bias	R2/R4	20%	–	–	–
Threshold bias	R3 (margin)	20%	50%	–	–
Obsolescence	R2 (reliability)	20%	50%	–	–
Obsolescence	R3 (margin)	40%	50%	–	–
Obsolescence	R4 (hybrid)	40%	–	–	–

Of 36 rule–mechanism–size combinations, 2 were unstable before compromise, 9 crossed $A = 1$, and 25 did not. Excluding the three validators with administrative signals (nine-member committee, Windows 7 as the third threshold-biased substitute) preserved the qualitative pattern and shifted crossovers upward (for example, the obsolescence crossover of R2 at $k = 3$ rose from 20% to 50%, and R3 and R4 did not cross $A = 1$ up to 50%). Figure S2 shows the median amplification for $k = 12$.

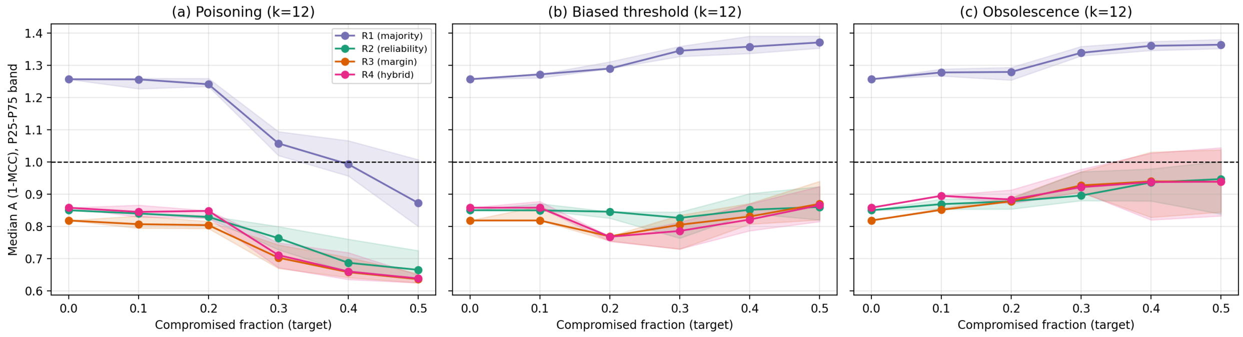

Figure S2: Supplementary grid: median amplification factor and uncertainty bands versus compromised fraction for the twelve-member committee.

These results cannot support committee-size recommendations. Seats were filled by repeating three identities, so the number of distinct adversaries did not grow with the compromised fraction; the effective redundancy of the committee was confounded with its nominal size; and the host and network validators contributed almost no signal to the IoT pool. The stress test in the main text uses instead a fixed seven-member committee in which every compromised seat is the degraded twin of a distinct source.

4 Deferral and Committee-Size Figure

Figure S3 shows the analysis with the twelve-member committee: (a) the trade-off between deferral and avoided false negatives, which assumed a perfectly accurate reviewer, and (b) MCC versus committee size without deferral. The reviewer-accuracy analysis of the main text is in Figure 10 of the article.

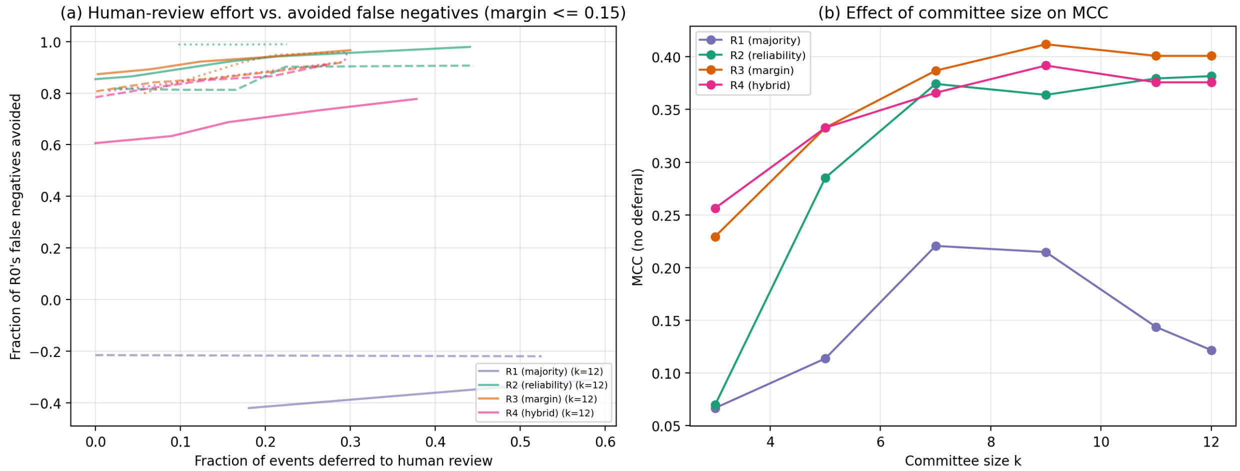

Figure S3: Deferral and committee-size analysis with the twelve-member committee (perfect-reviewer assumption). In panel (a), dotted, dashed and solid lines correspond to committees of 3, 7 and 12 validators.
